# Liver Endothelia Orchestrate MASH‐Associated Macrophage Zonation Through FSTL1‐ITGA4 Axis

**DOI:** 10.1002/advs.76410

**Published:** 2026-08-03

**Authors:** Lin Sun, Zhensheng Yue, Zhiqiang Fang, Hao Xu, Wei Du, Yuwei Ling, Jingjing Liu, Ping Song, Fei He, Juanli Duan, Lin Wang

**Affiliations:** ^1^ Department of Hepatobiliary Surgery Xijing Hospital Fourth Military Medical University Xi'an China; ^2^ Department of Ophthalmology Xi‐Jing Hospital Fourth Military Medical University Xi'an China

**Keywords:** fstl1, itga4, Kit, liver zonation, metabolic dysfunction‐associated steatohepatitis

## Abstract

**Background and Aims**: Liver zonation is fundamental to hepatic physiology. The zonal loss of Kit in liver sinusoidal endothelial cells (LSECs) during MASH suggests a role in niche maintenance, but its regulatory function is unclear. This study aimed to define how the zonated LSEC Kit controls macrophage distribution in MASH.

**Methods**: We used zonal LSEC isolation, endothelial‐specific knockout mice, spatial transcriptomics, and human data to dissect this axis.

**Results**: Pericentral LSEC Kit maintains homeostasis by suppressing FSTL1 via STAT2 phosphorylation. In MASH, Kit loss triggers centrilobular FSTL1 upregulation. The resulting FSTL1 gradient drives centrilobular accumulation of pro‐inflammatory ITGA4^+^ macrophages via NF‐κB. Inhibiting FSTL1 or ITGA4 restored macrophage zonation and ameliorated MASH.

**Conclusion**: The Kit/STAT2/FSTL1/ITGA4 axis is a master regulator of inflammatory zonation in MASH, revealing how zonal endothelial dysfunction drives spatial immune reorganization and offering a new framework for therapy.

## Graphical abstract

Liver inflammation in MASH fundamentally hinges on the newly defined Kit/STAT2/FSTL1/ITGA4 axis. Declining endothelial Kit collapses normal hepatic zonation, driving an aberrant FSTL1 gradient that recruits pathogenic macrophages. Strikingly, targeting FSTL1‐ITGA4 with anti‐VLA4 restores spatial immune homeostasis and halts disease progression, unveiling the strategic reversal of inflammatory zonation as a captivating therapeutic frontier.

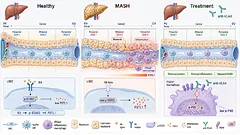

**Abbreviations**
α‐SMA: alpha‐smooth muscle actin
CDAA: choline‐deficient L‐amino‐defined
FSTL1: follistatin‐like 1
Fstl1‐KO: endothelial cell‐specific Fstl1 knockout
ITGA4: integrin α4
Kit‐KO: endothelial cell‐specific Kit knockout
LAMC1: laminin γ1
LSECs: liver sinusoidal endothelial cells
MAFLD: metabolic dysfunction‐associated fatty liver disease
MASH: metabolic dysfunction‐associated steatohepatitis
NAS: NAFLD activity score

## 1 Introduction

Metabolic dysfunction‐associated fatty liver disease (MAFLD) and its progressive form, metabolic dysfunction‐associated steatohepatitis (MASH), constitute a major global health burden characterized by a spectrum of hepatic damage that includes steatosis, inflammation, and fibrosis [1]. The pathogenesis of MASH involves complex cross‐talk among various liver cell types. Liver sinusoidal endothelial cells (LSECs), the most abundant non‐parenchymal cells, form a highly permeable barrier and are now recognized as key regulators of hepatic immune homeostasis. Their phenotypic and functional dysfunction is considered an early event in MASH pathogenesis [2, 3, 4]. Notably, LSECs exhibit remarkable zonal heterogeneity along the porto‐central axis, and distinct subpopulations are thought to influence the local microenvironment through compartmentalized secretion of specific factors [5]. Hepatic macrophages, including resident Kupffer cells and recruited monocyte‐derived macrophages, serve as central mediators of MASH‐related inflammation. Their spatial distribution within the hepatic lobule and their polarization state are critical determinants of disease progression and resolution [6]. However, the causal relationship between LSEC dysfunction and macrophage redistribution/inflammation in MASH has not been fully elucidated, and the molecular signals mediating this cross‐talk remain poorly defined.

In this study, we focus on follistatin‐like 1 (FSTL1), a secreted glycoprotein with context‐dependent roles in inflammation and immunity. Its function in the liver, particularly within LSECs, is largely unknown [7, 8, 9, 10]. Previous studies, including our own, have demonstrated that the receptor tyrosine kinase Kit is expressed in a zonal pattern by LSECs and that its expression is markedly reduced during the progression of MASH [11, 12, 13]. This zonally restricted loss of an endothelial marker suggests that Kit may contribute to maintaining a non‐inflammatory hepatic microenvironment, potentially by regulating the expression of downstream effector molecules.

Accordingly, we hypothesized that endothelial Kit loss in MASH disrupts the normal zonal distribution of hepatic macrophages and promotes their inflammatory activation through derepression of a key secreted factor. We identified FSTL1 as a primary candidate mediating the signal between LSECs and macrophages. To test this hypothesis, we used integrated approaches, including murine MASH models, in vitro cellular and molecular assays, and human clinical data analyses.

Our results delineate a novel paracrine signaling axis: injured LSECs in MASH drive macrophage inflammation via the Kit/STAT2/FSTL1 pathway. Mechanistically, endothelial Kit constitutively suppresses FSTL1 expression through the transcription factor STAT2. In MASH, Kit loss relieves this STAT2‐mediated repression, leading to marked upregulation and secretion of FSTL1. Secreted FSTL1 then binds directly to integrin α4 (ITGA4) receptors on macrophages, activating NF‐κB signaling. This cascade coordinately induces centrilobular macrophage accumulation and pro‐inflammatory polarization, thereby exacerbating MASH pathology. Our findings provide new insights into zonated intercellular communication in MASH and establish a strong rationale for developing therapies targeting the FSTL1‐ITGA4 axis.

## 2 Results

### 2.1 Spatial Heterogeneity of Hepatic Macrophages in MASH Mice

Previous studies have shown that liver‐resident macrophages, known as Kupffer cells, are predominantly enriched in the periportal region under physiological conditions [14, 15, 16]. However, in MASH, consistent with Guilliams et al. [17], spatial transcriptomics analysis (GSE248077) revealed significant liver macrophage accumulation around the central vein (CV) (Figure 1A). Cell–cell interaction analysis further revealed strong interactions between endothelial cells and macrophages (Figure 1B). In contrast, no obvious spatial heterogeneity was detected among other cell subtypes (Figure S1A). Notably, the interaction strength between endothelial cells and macrophages was particularly prominent in the overall cell–cell interaction analysis (Figure S1B). Moreover, in the MASH condition, bubble plot visualization of endothelial cell interactions with all cell types demonstrated that their communication with macrophages was the most significant (Figure S1C).

**FIGURE 1:**
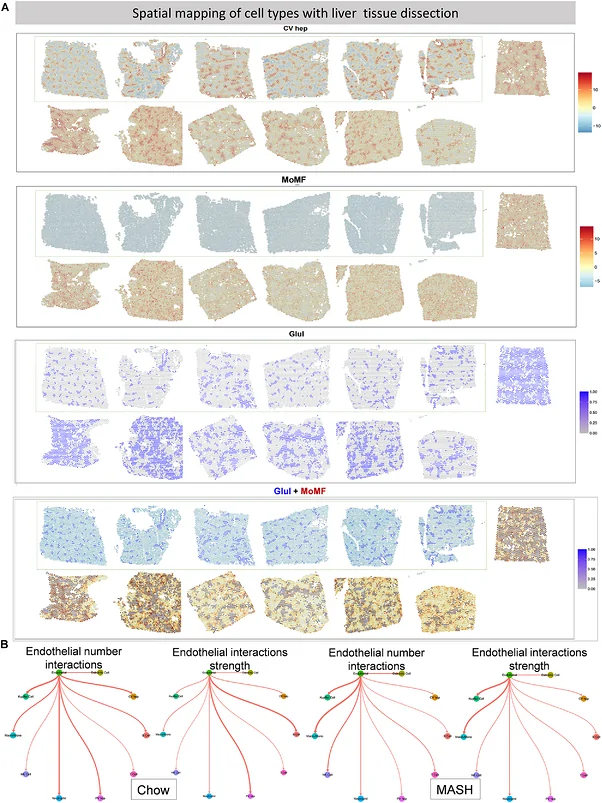
Spatial heterogeneity of hepatic macrophages in MASH mice. (A) Spatial transcriptomics analysis of liver sections from chow‑fed (n  =  6) and MASH diet‑fed (n  =  7) mice (GSE248077). The spatial distribution of central venous hepatocytes (CV hep; identified by marker genes Cyp2e1 and Glul) and monocyte‑derived macrophages (MoMFs) (S100a4, Ms4a4c, Lyz2, Itgam, Cx3cr1, Adgre1, Cd51, Ccr2, Trem2) is shown. An overlay of Glul expression and MoMF distribution is also provided. Color intensity represents the relative expression level of the corresponding marker genes per spot. (B) Cell–cell interaction networks highlighting the quantity (number of edges) and intensity (edge thickness) of communications involving endothelial cells, comparing livers from normal chow‐fed and MASH diet‐fed mice.

### 2.2 The Spatial Heterogeneity of MASH‐Associated Macrophages Is Associated With the Loss of Endothelial Kit in Liver Sinusoids

To investigate this phenomenon, we established a CDAA diet‐induced MASH model and collected liver tissues at 1, 2, 4, 8, and 10 weeks. Histological examination using hematoxylin and eosin (H&E) staining and glutamine synthetase (GS) immunohistochemistry revealed a progressive shift of steatosis from the periportal (PV) to the CV regions (Figure S2A).

LSECs, which represent the largest population of non‐parenchymal liver cells and are anatomically adjacent to Kupffer cells, were examined for their potential role in regulating macrophage spatial distribution. The functional implications of this asymmetric macrophage localization remain incompletely understood. Our previous work demonstrated zonal heterogeneity in LSEC Kit expression, with Kit^+^ LSECs predominantly located in the CV region and Kit^−^ LSECs in the PV region. Notably, Kit expression in CV‐associated LSECs progressively declined during MASH progression (Figure 2A; Figure S2A).

**FIGURE 2:**
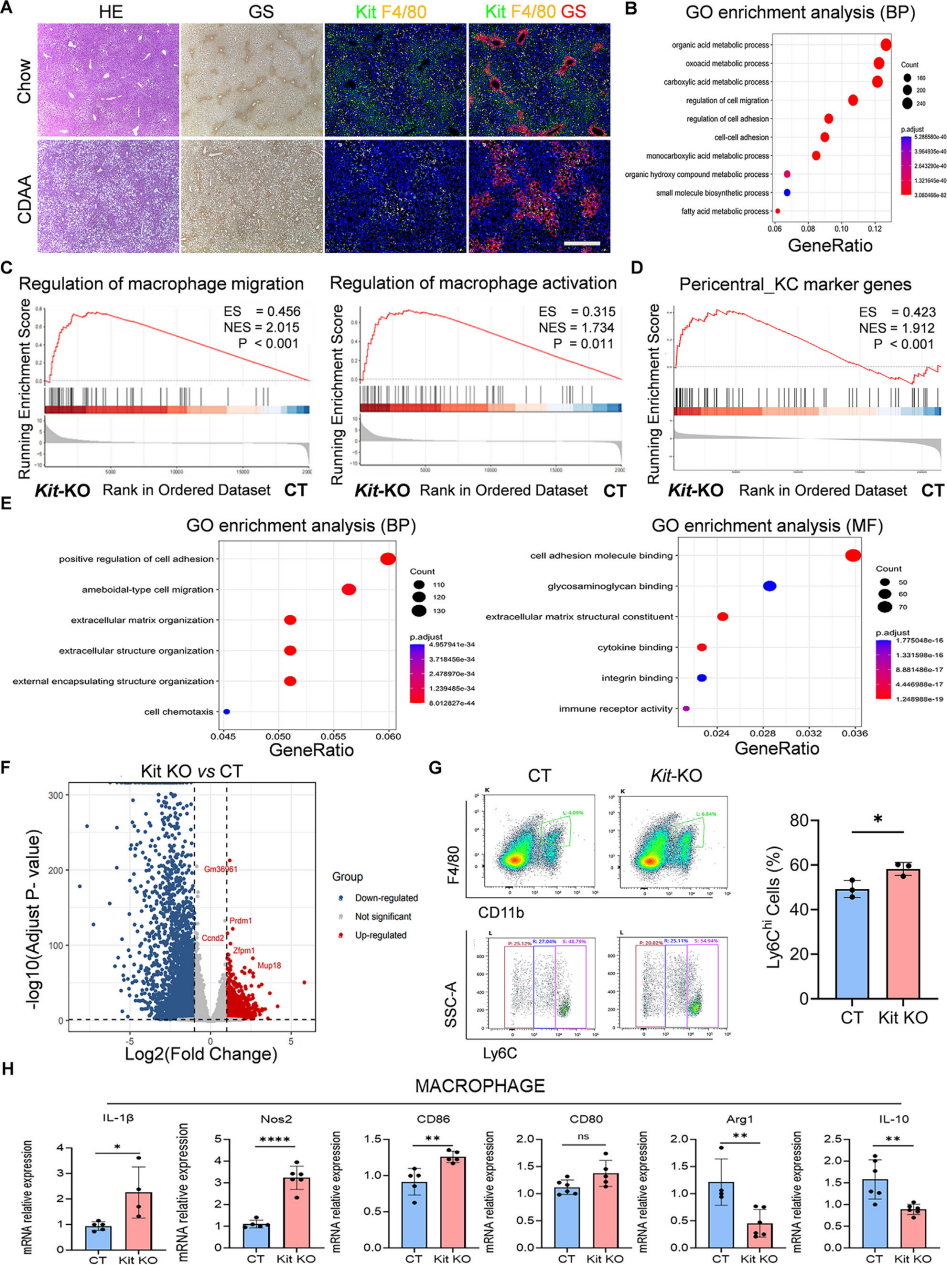
Macrophages Enrich in the Central Vein Region during MASH. (A) Liver sections from mice fed a CDAA diet for 10 weeks show enrichment of GS in the CV region (red) as demonstrated by H&E staining, GS immunohistochemistry, and GS immunofluorescence. H&E staining, GS immunohistochemistry, scale bars, 500 µm. Immunofluorescence co‐staining of F4/80^+^ cells (yellow), GS(red) and Kit (green) in LSECs showing enrichment in the CV; scale bars, 200 µm. (B and C) Functional enrichment analysis of differentially expressed genes (DEGs) LSECs from Kit KO mice and control mice, along with GSEA plots of pathways related to macrophage regulation. (D) GSEA plots of primary macrophages from Kit KO and control mice compared with CV region macrophages. (E) Functional enrichment analysis of DEGs in macrophages from Kit KO and control mice. (F) Volcano plot showing log_2_ fold changes in macrophages from Kit KO mice. (G) Representative flow cytometry plots of liver mononuclear cells from Kit KO and control mice. (H) Macrophage mRNA expression of indicated inflammatory genes relative to values in control mice. Data are presented as mean ± SEM. Statistical analysis was performed using Student's t‐test or multiple t‐tests (n = 3–6 mice per group). *p < 0.05, **p < 0.01, ***p < 0.001.

To investigate the role of endothelial Kit, we generated endothelial cell‐specific‐Kit knockout (Kit KO) mice and performed RNA sequencing (RNA‐seq) on LSECs isolated from both knockout and control mice. Gene Ontology (GO) analysis revealed several upregulated biological processes in Kit KO LSECs, including positive regulation of cell adhesion and cell migration (Figure 2B). Gene Set Enrichment Analysis (GSEA) further demonstrated enrichment of gene sets associated with macrophage migration and activation in Kit‐deficient endothelial cells (Figure 2C).

We then isolated primary hepatic macrophages from endothelial Kit KO mice and conducted RNA‐seq to evaluate the impact of endothelial Kit deletion on macrophage transcriptional profiles. To determine whether endothelial Kit influences macrophage localization, we reanalyzed a publicly available single‐cell RNA‐seq dataset GSE213165, which profiles CD45^+^ cells from the portal vein and central vein zones of the healthy mouse liver. Based on the relative proportions of periportal and centrilobular macrophages, we re‑clustered macrophage populations into two subsets, MP1 (PV‑like) and MP2 (CV‑like) (Figure S2B). GSEA using these subset signatures on RNA‑seq data from endothelial Kit KO macrophages showed significant enrichment of the MP2 signature, but not the MP1 signature (Figure 2D; Figure S2C). Consistently, GO analysis revealed enrichment of chemotaxis, migration, and adhesion pathways in Kit KO macrophages (Figure 2E). Differentially expressed genes and immune/chemokine signaling gene expression patterns are presented in Figure 2F and Figure S2D.

We further characterized phenotypic alterations in macrophages following endothelial Kit knockout using flow cytometry. The analysis revealed a shift toward a pro‐inflammatory Ly6C^high^ macrophage phenotype in knockout mice (Figure 2G). Quantitative PCR (qPCR) of primary macrophages isolated from endothelial Kit KO mice confirmed the upregulation of key pro‐inflammatory genes, corroborating the observed pro‐inflammatory transition (Figure 2H). Together, these results demonstrate that endothelial Kit loss not only enriches a CV macrophage signature with enhanced chemotaxis and migration but also drives a shift toward a pro‐inflammatory Ly6C^high^ phenotype, collectively establishing a pro‐inflammatory and spatially dysregulated macrophage state.

### 2.3 FSTL1 Expression Is Upregulated in MASH and Exhibits Zonal Heterogeneity in both Human Patients and Diet‐Induced MASH Mice

We next sought to identify potential mediators through which endothelial Kit may regulate macrophage function, we cross‐referenced the mouse liver secretome dataset [18] with highly secreted factors derived from PV Kit^−^ LSECs isolated from chow‐fed mice (Figure 3A). This comparison yielded a shortlist of 20 candidate secretory factors (Figure 3B). Among these, FSTL1‐a secreted glycoprotein previously implicated in macrophage biology but with undefined functions in LSECs, was selected for further investigation. Immunoblot analysis confirmed its high expression in PV LSECs of chow‐fed mice (Figure 3C). To examine FSTL1 expression dynamics during MASLD progression, we performed immunofluorescence staining on liver sections from CDAA diet‐fed and chow‐fed mice. Notably, FSTL1 expression was increased in the CV region of CDAA‐fed mice and exhibited clear zonal heterogeneity (Figure 3D). Analysis of a publicly available spatial transcriptomics dataset further supported these findings, demonstrating elevated FSTL1 expression in MASH livers, with higher levels in the CV region compared to the PV region (Figure S3A).

**FIGURE 3:**
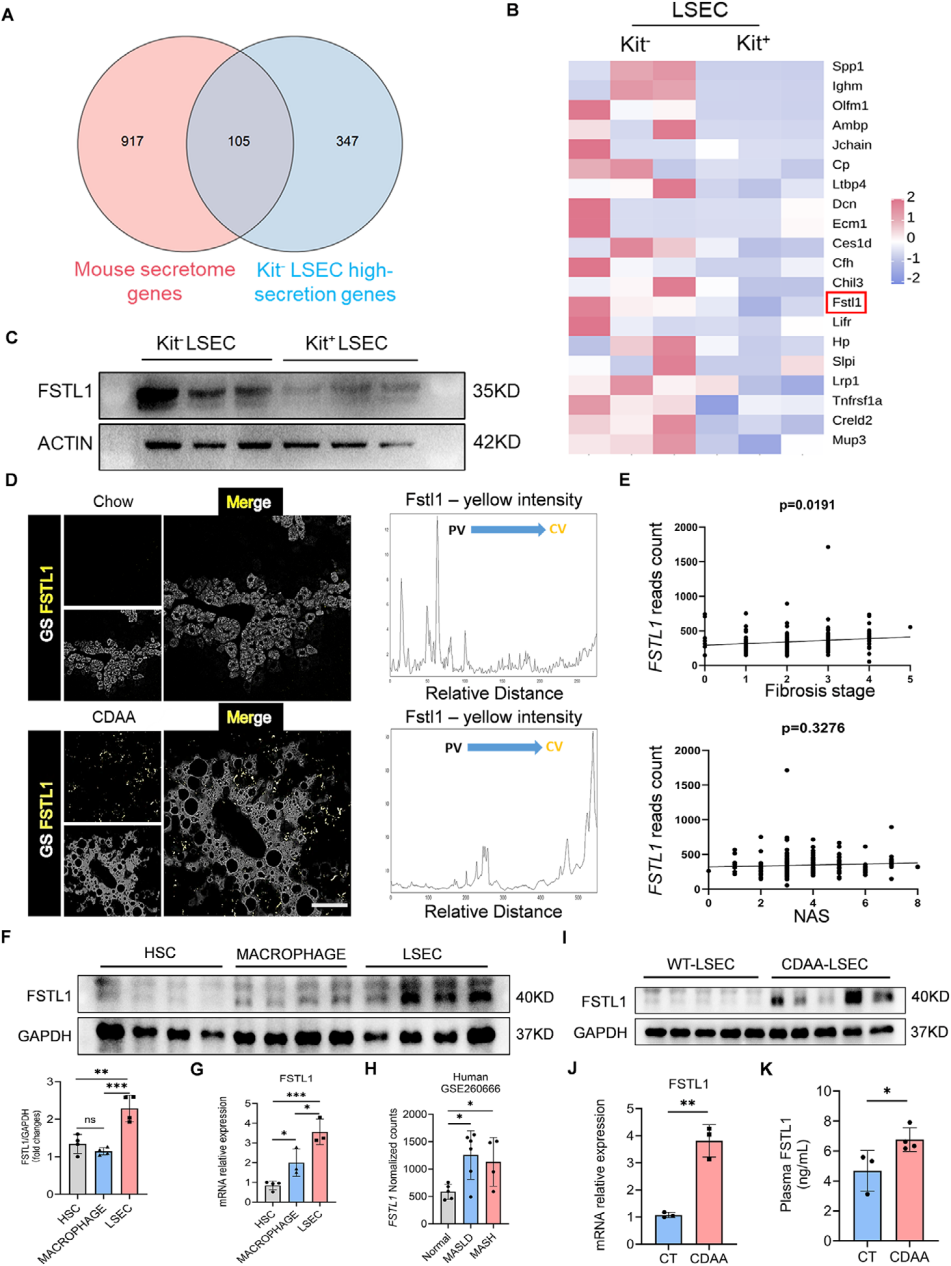
FSTL1 expression is upregulated in liver tissues from MASH patients and diet‐induced MASH mice. (A) Venn diagram showing the overlap between the murine liver secretory expression profile from a public database and the secretory profile of cultured Kit^−^ LSECs. (B) Heatmap displaying the top 20 overlapping secretory factors. (C) Immunoblot of FSTL1 from Kit^−^ LSECs and Kit^+^ LSECs of wild‐type mouse livers. (D) Representative immunofluorescence co‐staining images of GS (white) and FSTL1 (yellow) in livers from CDAA‐fed and control mice; scale bars, 50 µm. Left: Representative immunofluorescence images of FSTL1 and GS expression in livers from mice fed a normal diet and a CDAA diet. Scale bars, 50 µm. Right: The graph shows the intensity of FSTL1 staining from the PV to the CV. Data are presented as mean fluorescence intensity ± s.e.m. (n = 5 biological replicates). (E) Correlation analysis of hepatic FSTL1 expression with the NAS and fibrosis stage in MASH patients from the GSE193084 dataset (n = 213). (F and G) FSTL1 protein expression levels (F) and mRNA expression levels detected by qPCR (G) in primary HSCs, macrophages, and LSECs from C57BL/6J mice. (H) FSTL1 mRNA expression levels in human liver samples from the GSE260666 dataset. (I) FSTL1 protein expression in primary LSECs isolated from CDAA‐induced and control mice. (J) Hepatic FSTL1 mRNA and protein expression levels in control and MASH mice. (K) Serum FSTL1 levels in control and MASH mice. Data are presented as mean ± SEM. Statistical analysis was performed using Student's t‐test or multiple t‐tests one‐way ANOVA. *p < 0.05, **p < 0.01, ***p < 0.001. For all animal studies, n = 3–6 per group.

Consistent with these results, immunoblot analysis of liver tissues from CDAA diet‐fed mice confirmed increased FSTL1 protein expression (Figure S3B). Furthermore, analysis of a human cirrhosis dataset (GSE193084) revealed a positive correlation between FSTL1 expression and fibrosis stage (Figure 3E).

To determine the cell type‐specific expression of FSTL1 within the liver, we isolated primary LSECs, macrophages, and hepatic stellate cells. Both transcriptional and protein analyses demonstrated that FSTL1 expression was highest in LSECs compared with the other non‐parenchymal cell types (Figure 3F,G). We next analyzed FSTL1 mRNA expression in human liver biopsies from the Gene Expression Omnibus (GEO) dataset GSE260666 and found a significant upregulation in patients with MAFLD and MASH compared with normal livers (Figure 3H).

Finally, reanalysis of our previous LSEC transcriptome data (volcano plot in Figure S3C), together with Western blot and qPCR results from LSECs isolated from chow‐fed and CDAA diet‐fed mice, consistently confirmed the upregulation of FSTL1 in MASH‐associated LSECs (Figure 3I,J). Additionally, enzyme‐linked immunosorbent assay (ELISA) measurements showed elevated serum FSTL1 levels in MASH mice (Figure 3K). Collectively, these findings suggest that FSTL1 may play an important role not only in hepatic pathophysiology but also systemically during MASH progression.

### 2.4 Endothelial Cell‐Specific Deletion of FSTL1 Ameliorates Injury in a MASH Model

To determine whether endothelial FSTL1 influences other hepatic cell types, particularly macrophages, we generated endothelial cell‐specific, inducible FSTL1 knockout (FSTL1 KO) mice by crossing Cdh5^CreERT^ with Fstl1^flox/flox^ mice (Figure 4A).

**FIGURE 4:**
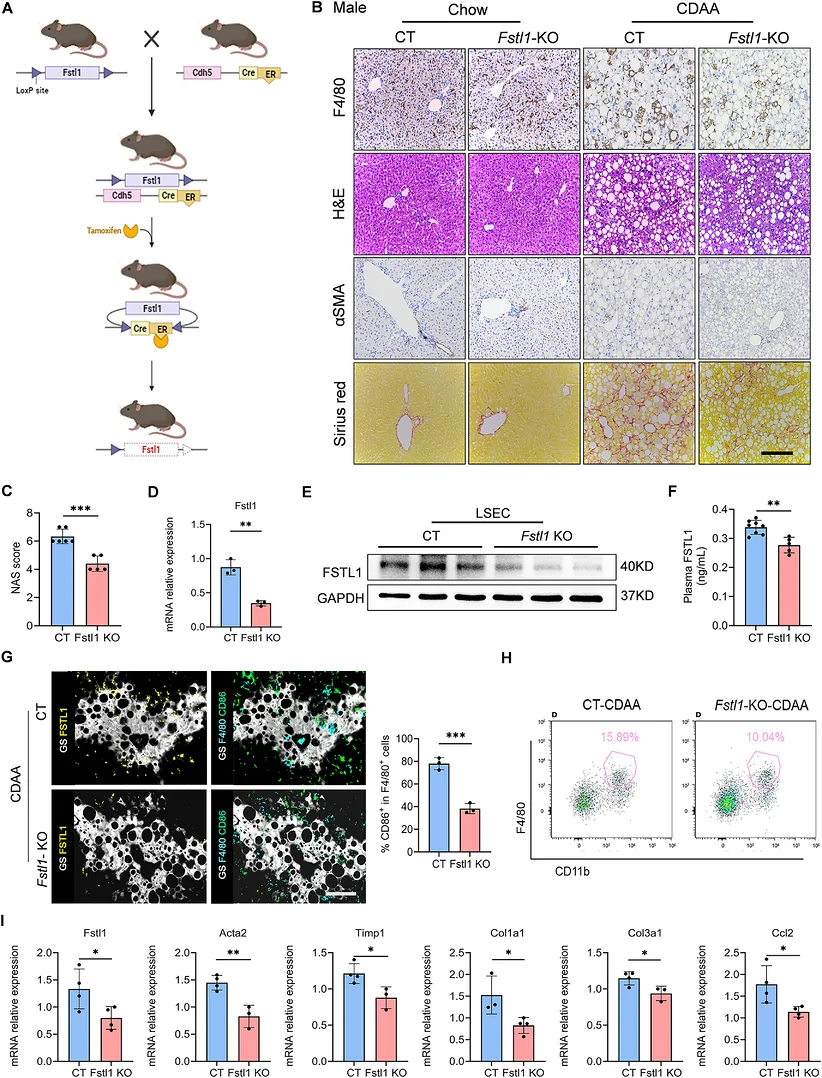
Endothelial‐specific FSTL1 knockout attenuates CDAA diet‐induced liver injury, inflammation, and fibrosis. (A) Schematic diagram of the experimental design for endothelial‐specific FSTL1 knockout. (B) Representative images of H&E, F4/80, and α‐SMA immunohistochemistry, as well as Sirius Red staining. Scale bars, 100 µm. (C) Histological scoring of liver tissues. (D–F) Hepatic FSTL1 mRNA (D) and protein levels (E), and serum FSTL1 levels (F). (G) Immunofluorescence images of FSTL1, GS and CD68, F4/80, GS in liver sections. Scale bars, 50 µm. (H) Representative flow cytometry plots of non‐parenchymal cells from the livers of CDAA‐fed FSTL1 KO and control mice. (I) Hepatic mRNA expression levels of Fstl1, Acta2, Timp1, Col1a1, Col3a1, and Ccl2 as determined by qPCR. Data are presented as mean ± SEM. Statistical analysis was performed using Student's t‐test or multiple t‐tests. *p < 0.05, **p < 0.01, ***p < 0.001 (biological replicates n = 3–8).

Under normal chow diet conditions, FSTL1 KO mice exhibited no apparent phenotypic abnormalities. However, after 10 weeks on a CDAA diet, FSTL1 KO mice displayed a lower NAFLD activity score (NAS), reduced hepatic steatosis, and a significant decrease in F4/80^+^ macrophages compared with control littermates (Figure 4B,C). Knockout efficiency was confirmed by a marked reduction in hepatic Fstl1 mRNA expression (Figure 4D) and undetectable FSTL1 protein levels in primary LSECs isolated from these mice (Figure 4E). Serum FSTL1 concentrations were also significantly lower in FSTL1 KO mice (Figure 4F).

Furthermore, immunofluorescence staining revealed reduced monocyte infiltration and a lower proportion of CD86^+^F4/80^+^ M1‐like macrophages in the livers of FSTL1 KO mice (Figure 4G). Flow cytometric analysis of non‐parenchymal liver cells from CDAA‐fed mice further corroborated the reduction in F4/80^+^CD11b^+^ macrophage populations (Figure 4H). Consistently, whole‐liver mRNA expression profiling revealed downregulation of key fibrotic and inflammatory genes, including Ccl2 (Figure 4I).

Because FSTL1, as a follistatin‐related protein, may exhibit ovarian‐associated expression, we specifically examined female mice to exclude potential sex bias. Endothelial FSTL1 deficiency provided consistent protection against CDAA diet‐induced MASH in female mice (Figure S4A), which was accompanied by a reduction in CV‐associated macrophages (Figure S4B), lower NAS values, and decreased serum FSTL1 levels (Figure S4C,D), along with a significant reduction in serum ALT levels (Figure S4E). Together, these results demonstrate a sex‐independent, pathogenic role for endothelial‐derived FSTL1 in promoting MASH progression.

Finally, to investigate the influence of sex on FSTL1 expression, we compared the phenotypes and hepatic FSTL1 expression levels between male and female mice fed a CDAA diet for 10 weeks. Observations revealed that female mice exhibited slightly milder hepatic steatosis and fibrosis compared to males, a phenomenon consistent with previously published studies demonstrating that female mice are significantly resistant to these pathological changes(Figure S5A). Subsequently, we analyzed FSTL1 expression levels in male and female mice by western blotting and compared FSTL1 expression differences between female mice on a CDAA diet and those on a normal diet. The results showed that although FSTL1 expression in female mice on the CDAA diet was slightly lower than in males, the difference was not statistically significant(Figure S5B). However, compared to the normal diet group, hepatic FSTL1 expression was significantly increased in female mice on the CDAA diet, a trend consistent with the observations in the male group (Figure S5C).

### 2.5 FSTL1 Directly Promotes Pro‐Inflammatory Polarization and Migration of Macrophages In Vitro

The direct effects of FSTL1 on macrophages were examined using a series of in vitro functional assays. Because FSTL1 is a secreted glycoprotein, we first treated THP‐1‐derived macrophages with recombinant human FSTL1 (rhFSTL1) for 24 h. Flow cytometric analysis revealed increased intracellular expression of the pro‐inflammatory cytokines TNF‐α and IL‐1β in rhFSTL1‐treated cells (Figure S6A). Consistently, mRNA levels of key inflammatory genes were elevated, indicating a shift toward an M1‐like pro‐inflammatory phenotype (Figure S6B).

Next, we overexpressed FSTL1 in human umbilical vein endothelial cells (HUVECs) and co‐cultured them with THP‐1 cells for 48 h. Successful FSTL1 overexpression in HUVECs was confirmed at both the transcript and protein levels (Figure S6C,D). A transwell migration assay demonstrated that THP‐1 cell migration was significantly enhanced when co‐cultured with FSTL1‐overexpressing HUVECs compared with control cells (Figure S6E).

To specifically determine the role of endothelial‐secreted FSTL1, we collected conditioned medium from FSTL1‐overexpressing HUVECs and applied it directly to THP‐1 macrophages. Addition of an FSTL1‐neutralizing polyclonal antibody to the conditioned medium effectively abolished the pro‐inflammatory effects. This was evidenced by increased expression of anti‐inflammatory markers (ARG1 and CD163) and decreased expression of pro‐inflammatory markers (TNF‐α and NOS2) in THP‐1 cells compared with those treated with control IgG (Figure S6F). Collectively, these findings demonstrate that endothelial‐derived FSTL1 directly promotes macrophage migration and strongly drives their polarization toward a pro‐inflammatory phenotype.

### 2.6 FSTL1 Regulates Macrophage M1 Polarization and Migration via Interaction With Integrin α4

To elucidate the molecular mediators underlying FSTL1's effects on macrophages, we conducted a STRING protein–protein interaction network analysis. This analysis revealed that FSTL1‐a secreted, cysteine‐rich glycoprotein structurally similar to SPARC‐may interact with laminin γ1 (LAMC1) and the ITGA4 receptor, among other partners (Figure 5A). Extracellular matrix proteins frequently exhibit high affinity for cell surface receptors, particularly integrins, which serve as key adhesion and signaling molecules on immune cells. Integrins function as critical bridges linking the intracellular cytoskeleton to the extracellular matrix, thereby mediating cell–environment interactions [19]. Analysis of publicly available transcriptomic datasets indicated increased ITGA4 expression predominantly in macrophages from MASH livers (Figure S7A,B). This finding was corroborated by our spatial transcriptomics data, which further demonstrated upregulated expression of Itga4, Ccl2, and Cd86, alongside downregulated Marco expression in MASH conditions (Figure S7C). These collective data led us to hypothesize that FSTL1 may signal through ITGA4 on the macrophage surface.

**FIGURE 5:**
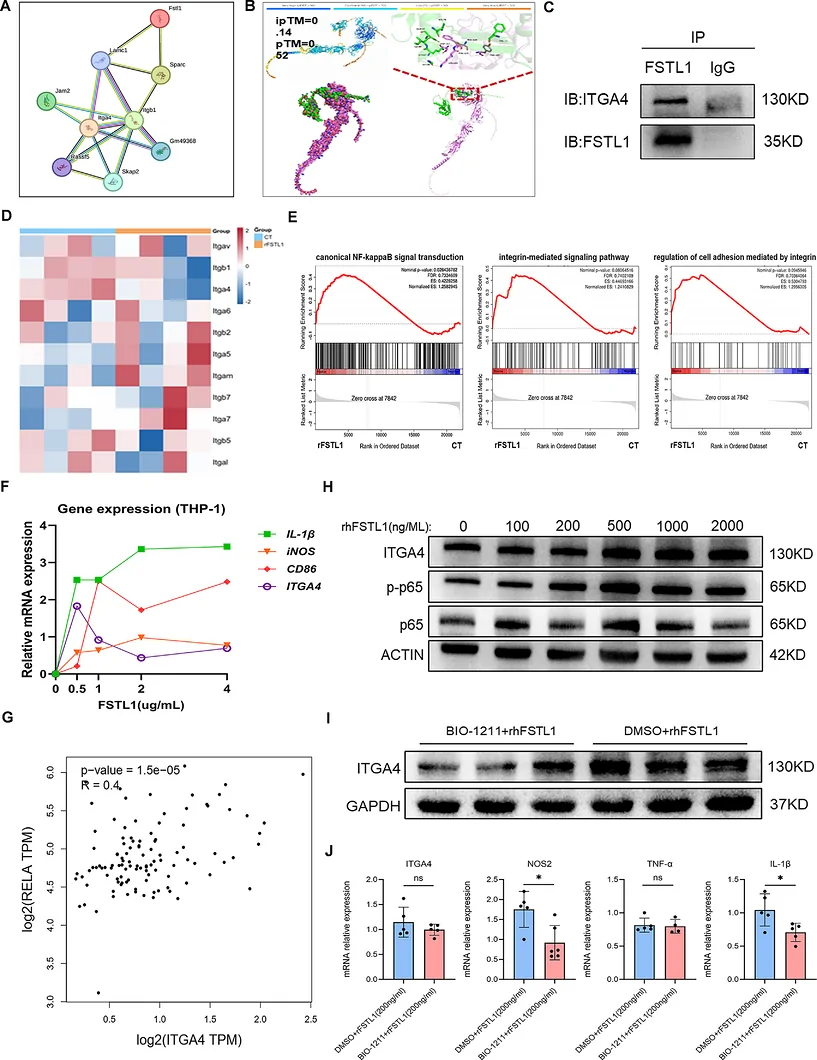
Integrin α4 expression is increased in MASH livers and activated macrophages, and FSTL1 binds to integrin α4 on activated macrophages. (A) Protein–protein interaction network between FSTL1 and ITGA4 from the STRING database. (B and C) Analysis of the predicted binding potential between FSTL1 and ITGA4 by AlphaFold, molecular docking model, and co‐immunoprecipitation immunoblot analysis of their interaction. (D) Heatmap of RNA‐seq data showing expression of integrin‐related molecules in RAW264.7 cells treated with or without recombinant FSTL1 (rFSTL1). (E) GSEA of enriched pathways in RAW264.7 cells treated with or without rFSTL1. (F) qPCR analysis of inflammatory factor expression in THP‐1 cells treated with varying concentrations of recombinant human FSTL1 (rhFSTL1) for 24 h. (G) Correlation analysis between ITGA4 and RELA (p65) mRNA levels from the GEPIA database. (H) Immunoblot analysis of NF‐κB pathway‐related molecules in lysates from THP‐1 cells treated with different concentrations of rhFSTL1 for 24 h. (I and J) THP‐1 cells were pre‐treated with BIO‐1211 for 4 h followed by rhFSTL1 stimulation for 24 h. (I) Immunoblot analysis of ITGA4 expression and (J) qPCR analysis of immune‐related gene expression in cell lysates. Data are presented as mean ± SEM. Statistical analysis was performed using Student's t‐test or multiple t‐tests. *p < 0.05, **p < 0.01, ***p < 0.001 (n = 3–6).

We first evaluated the potential interaction between FSTL1 and ITGA4 using AlphaFold structural prediction, which revealed plausible binding interfaces between the two proteins (Figure 5B). This interaction was experimentally validated by co‐immunoprecipitation analysis, confirming that FSTL1 directly binds to ITGA4 on macrophages (Figure 5C).

Subsequent RNA‐seq analysis of RAW264.7 cells treated with recombinant FSTL1 revealed that the heatmap displayed expression changes of integrin family‐related genes, with ITGA4 being significantly downregulated‐potentially due to conformational alterations (Figure 5D). GSEA further demonstrated significant enrichment of integrin signaling and inflammation‐related pathways (Figure 5E). Consistently, GSEA of primary macrophages from Kit KO mice also showed enrichment of integrin‐associated signaling pathways (Figure S7D).

We next investigated the functional role of ITGA4 in FSTL1‐induced macrophage inflammation. Contrary to our previous RNA‐seq data, treatment of THP‐1 cells with varying concentrations of recombinant FSTL1 significantly upregulated ITGA4 expression, with a concomitant increase in the expression of related inflammatory genes (Figure 5F). A heatmap of differentially expressed genes in Kit KO primary macrophages further supported ITGA4 involvement in chemokine signaling and NF‐κB activation (Figure S7E). Correlation analysis using the GEPIA database revealed a positive association between ITGA4 and RELA (p65) mRNA expression (Figure 5G). Moreover, treatment of THP‐1 cells with increasing concentrations of rhFSTL1 resulted in a dose‐dependent upregulation of ITGA4 and enhanced NF‐κB activation (Figure 5H).

To confirm that FSTL1 exerts its effects specifically through ITGA4, THP‐1 cells were pretreated with the ITGA4‐specific inhibitor BIO‐1211. This pretreatment effectively suppressed ITGA4 protein expression (Figure 5I) and significantly attenuated FSTL1‐induced pro‐inflammatory gene expression (Figure 5J). Collectively, these findings demonstrate that FSTL1‐induced macrophage pro‐inflammatory polarization is mediated through the ITGA4 signaling axis.

### 2.7 Endothelial FSTL1 Deletion Attenuates MASH‐Induced Enrichment of ITGA4‐Expressing Macrophages in the Central Vein Region

We next examined the impact of endothelial FSTL1 deletion on ITGA4^+^ macrophages in vivo in the MASH model. Liver sections from CDAA diet‐fed control and FSTL1 KO mice were subjected to multiplex immunofluorescence staining for CD31, FSTL1, F4/80, ITGA4, and GS. Quantitative analysis of defined regions of interest within the CV and PV areas was performed to enumerate total F4/80^+^ macrophages and the subset co‐expressing ITGA4 (Figure 6A,B). This analysis revealed a significant reduction in both the total macrophage population and the number of ITGA4^+^ macrophages in FSTL1 KO mice compared with controls (Figure 6C).

**FIGURE 6:**
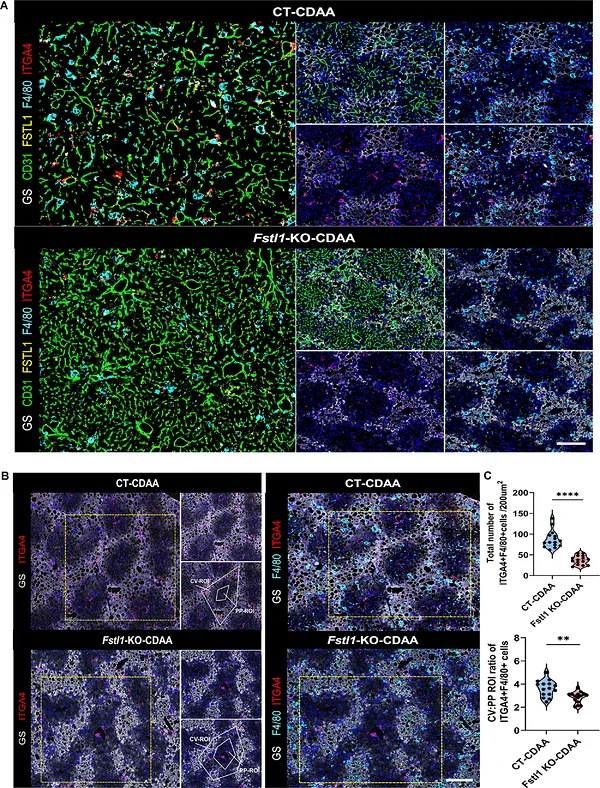
ITGA4^+^ macrophages are enriched in the central vein region, an effect disrupted by endothelial FSTL1 knockout. (A) Representative immunofluorescence images showing the localization of CD31 (green), F4/80 (cyan), ITGA4 (red), FSTL1 (yellow), and GS (white) in livers of CDAA‐fed FSTL1 KO and control mice. Scale bars,100 µm. (B) Co‐localization of ITGA4 with GS, and triple‐localization of F4/80, ITGA4, and GS in the two groups. The right panels illustrate the method for quantifying the region of interest (ROI). Scale bars, 200 µm. (C) Quantification of the ROI analysis for both groups. The ROIs were selected by randomly choosing fields of view where the CV region fully encircled a portal vein. For each sample, three specific ROIs meeting this anatomical criterion were uniformly selected. The individual data points in the scatter plot represent measurements from these ROIs (biological replicates, n = 5). Data pooled from two independent experiments are presented as the median ± quartile range. Statistical significance was determined using the two‐tailed Mann‐Whitney U test, with significance levels denoted as **p < 0.01 and ****p < 0.0001.

To further assess spatial localization of ITGA4, co‐staining with GS confirmed reduced ITGA4 expression specifically within the CV region of FSTL1 KO mice (Figure S8A). Immunoblot analysis of whole‐liver lysates corroborated these results, showing markedly decreased ITGA4 protein levels and reduced expression of the fibrotic marker α‐smooth muscle actin (α‐SMA) in FSTL1 KO mice (Figure S8B).

To validate our hypothesis that endothelial Kit influences macrophage zonation, we analyzed liver tissues from chow‐fed endothelial‐specific Kit KO mice and corresponding controls. H&E and immunohistochemical (F4/80) analyses revealed an exacerbated inflammatory phenotype following Kit deletion. Moreover, immunofluorescence co‐staining for GS and ITGA4 demonstrated increased ITGA4 expression within the CV region of Kit KO mice (Figure S8C). This pattern was further observed in CDAA diet‐fed mice, which exhibited increased ITGA4^+^ macrophages in the CV region compared with chow‐fed controls (Figure S8D).

Quantitative immunofluorescence analysis (F4/80, ITGA4, GS) in chow‐fed Kit KO and control mice demonstrated increased numbers of F4/80^+^, ITGA4^+^, and double‐positive macrophages in Kit KO livers (Figure S8E). Consistently, immunoblot analysis of primary macrophages isolated from these mice confirmed that endothelial Kit deletion upregulates ITGA4 expression in macrophages (Figure S8F).

### 2.8 Recombinant Mouse FSTL1 Administration Exacerbates the MASH Phenotype, Which Can be Rescued by an Integrin α4 Antibody

To further evaluate the direct effect of elevated hepatic FSTL1 on liver inflammation in mice, we administered intraperitoneal injections of recombinant mouse FSTL1 to normal diet‐fed mice and monitored changes in hepatic inflammatory phenotypes. Recombinant FSTL1 was then co‐administered with either an ITGA4 antibody (anti‐VLA4) or rat‐derived IgG as an isotype control. In a separate experimental arm, CDAA diet‐fed mice received intraperitoneal injections of anti‐VLA4 to assess its effects on liver pathology (Figure 7A). In the normal diet group, H&E staining and immunohistochemical analysis revealed a marked increase in hepatic inflammatory cell infiltration following FSTL1 administration, which was significantly attenuated by anti‐VLA4 treatment (Figure 7B). No significant differences were observed in body weight or liver‐to‐body weight ratio among the groups (Figure 7C). Serum levels of ALT and AST, which were elevated by FSTL1 treatment, were markedly reduced following anti‐VLA4 co‐administration (Figure 7D). Western blot and qPCR analyses demonstrated that FSTL1 strongly induced hepatic inflammation, which was effectively reversed by ITGA4 inhibition using anti‐VLA4 (Figure 7E,F,G).

**FIGURE 7:**
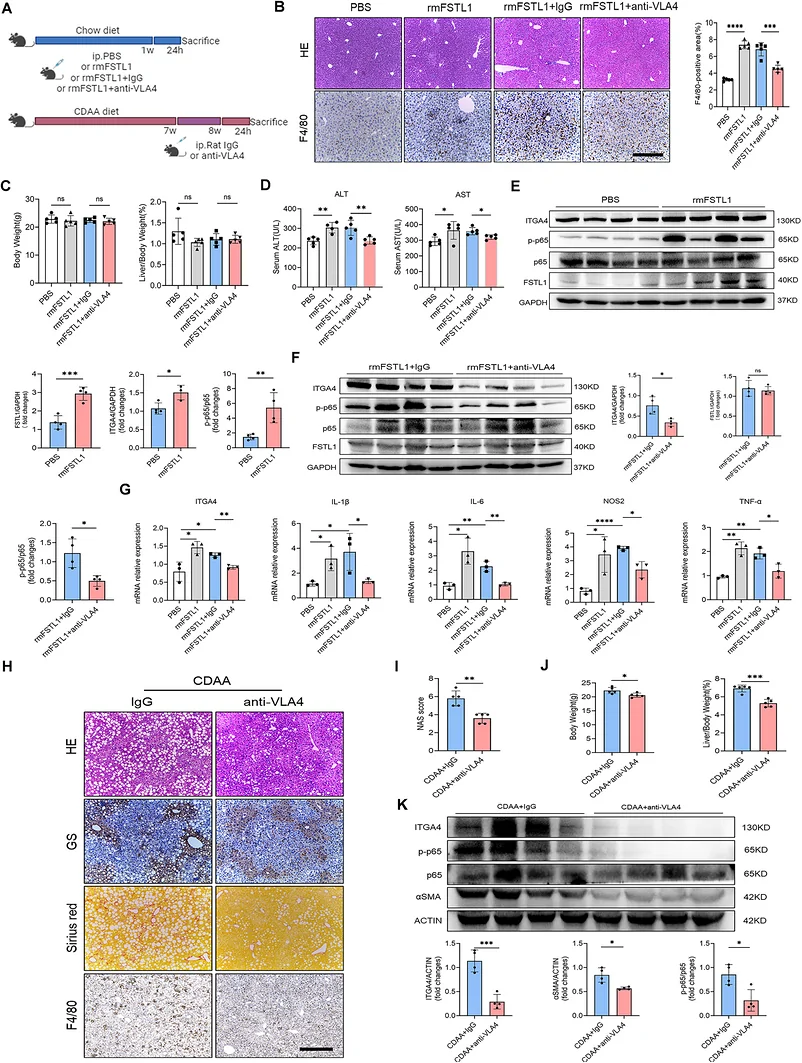
The inflammatory phenotype induced by recombinant mouse FSTL1 administration in mice is significantly ameliorated by ITGA4 blockade. (A) Schematic diagram of the experimental design: administration of FSTL1 with or without anti‐VLA4 antibody under normal chow diet, and administration of anti‐VLA4 antibody in the CDAA model. (B) Representative H&E and F4/80 immunohistochemistry staining of mouse livers with corresponding quantitative analysis. Scale bars, 200 µm. (C) Body weight and liver‐to‐body weight ratio. (D) Serum AST and ALT levels. (E and F) Western blot analysis of indicated proteins in liver tissues. (G) Relative mRNA expression levels of inflammation‐related genes in mouse livers as determined by quantitative RT‐PCR. (H) Representative H&E, Sirius Red staining, and GS, F4/80 immunohistochemistry of livers from CDAA‐fed mice. Scale bars, 200 µm. (I) NAS of livers. (J) Body weight and liver‐to‐body weight ratio in CDAA‐fed groups. (K) Western blot analysis of indicated proteins in liver tissues from CDAA‐fed groups. Data are presented as mean ± SEM. Statistical analysis was performed using Student's t‐test or multiple t‐tests. *p < 0.05, **p < 0.01, ***p < 0.001 (n = 3–5).

Given the key role of FSTL1 in activating integrin α4 and its diverse biological functions in the pathogenesis of MASLD, we further investigated the contribution of ITGA4 to FSTL1‐induced hepatic inflammation and fibrosis. CDAA diet‐fed mice were treated for 7 days with daily intraperitoneal injections of anti‐VLA4 or rat‐derived IgG during the final week of the 7‐week dietary regimen. Anti‐VLA4 treatment significantly ameliorated CDAA diet‐induced hepatic fibrosis and inflammation (Figure 7H), improved NAS, and reduced both body weight and liver‐to‐body weight ratio (Figure 7I,J). Western blot analysis confirmed the downregulation of fibrosis‐ and inflammation‐associated markers in anti‐VLA4‐treated mice (Figure 7K). Collectively, these findings demonstrate that FSTL1 exacerbates hepatic inflammation through ITGA4 activation and that pharmacological inhibition of integrin α4 effectively mitigates MASH progression.

To investigate the potential influence of sex on treatment efficacy, female mice fed a CDAA diet for 7 weeks were administered anti‐VLA4 or control IgG. Histological examination using H&E, Sirius Red, and immunohistochemistry for GS and F4/80 revealed that anti‐VLA4 treatment significantly alleviated the MASH phenotype in female mice, consistent with the results observed in males (Figure S9A). To further investigate whether the weight loss observed in male mice receiving anti‐VLA4 treatment reflected increased systemic stress rather than improved metabolic health, we subjected both groups of male mice to metabolic cage monitoring. We first assessed whether the weight reduction was associated with metabolic suppression by analyzing energy expenditure (EE). The key finding was that EE remained stable in the anti‐VLA4‐treated group compared to the control group (Figure S9B). This result is critical: it indicates that the weight loss in the treatment group did not arise from a catabolic wasting state (which typically leads to reduced EE), but rather reflected an active metabolic adaptation involving enhanced lipid oxidation. Consistent with the EE data, oxygen consumption (VO_2_) and carbon dioxide production (VCO_2_) in the anti‐VLA4‐treated group were comparable to those in the control group, despite the observed weight loss (Figure S9C). Importantly, the changes in body weight were independent of significant alterations in food or water intake, as no differences were detected between the two groups (Figure S9D). This suggests that overall metabolic activity per unit body mass was actually enhanced, further supporting the notion of a therapeutic metabolic shift rather than treatment‐induced toxicity.

### 2.9 Short‐Term, Low‐Dose Natalizumab Treatment Ameliorates the MASH Phenotype and Restores Macrophage Zonation in Mice

To evaluate whether natalizumab‐a clinically approved monoclonal antibody targeting ITGA4 and used for the treatment of inflammatory bowel disease‐could exert therapeutic effects in a MASH model under a short‐term, low‐dose regimen, we administered the drug to mice after 7 weeks on a CDAA diet. Mice received tail vein injections of humanized natalizumab or control human IgG (Figure 8A).

**FIGURE 8:**
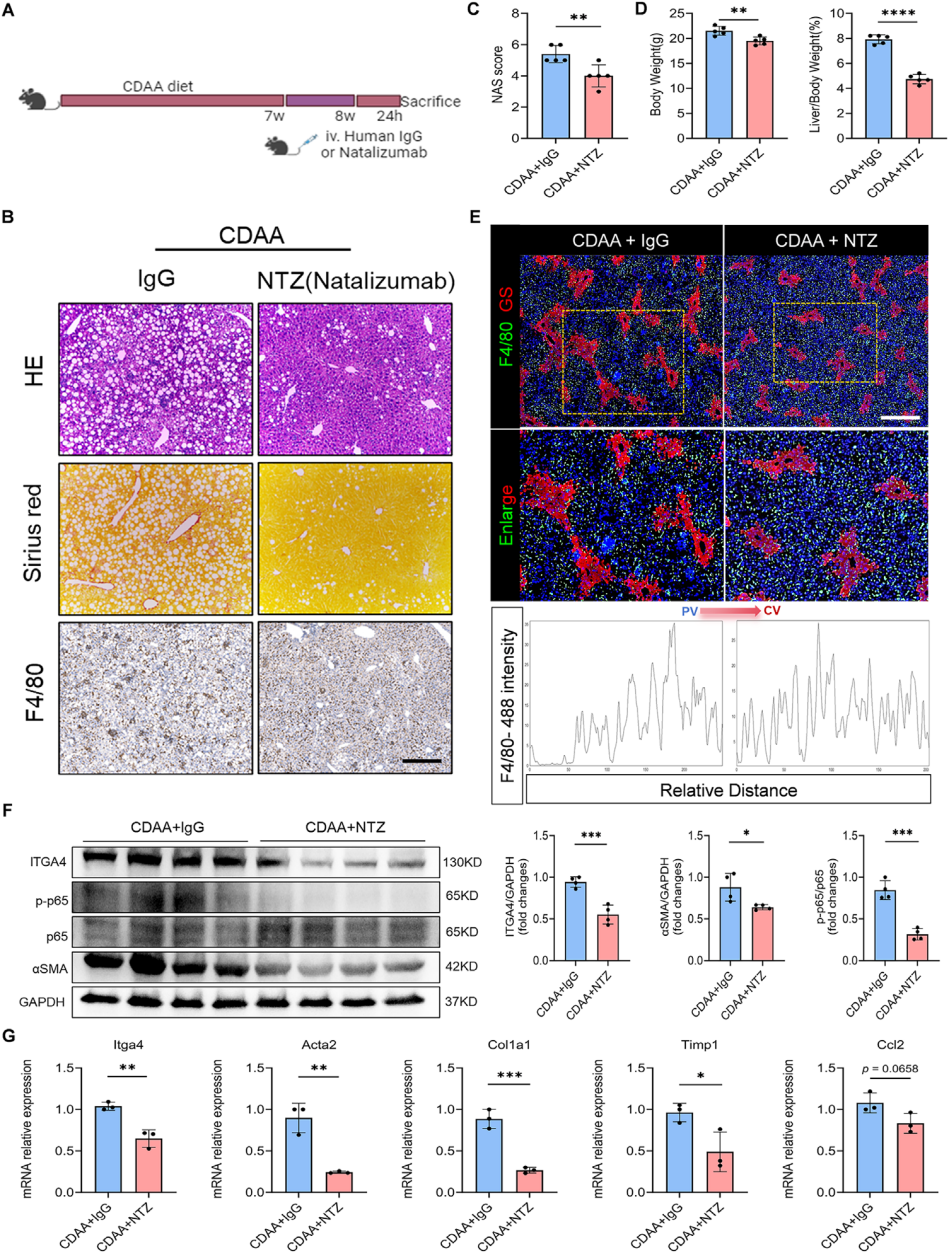
Short‐term, low‐dose natalizumab treatment alleviates MASH phenotypes in mice. (A) Experimental timeline of natalizumab administration in the CDAA diet‐induced MASH model. (B) Representative liver sections showing H&E staining, Sirius Red staining, and F4/80 immunohistochemistry. Scale bars, 200 µm. (C) NAS of liver tissues. (D) Body weight and liver‐to‐body weight ratio. (E) Representative immunofluorescence images and corresponding line graphs quantifying fluorescence intensity for F4/80 and GS in liver tissues from the two groups. Scale bars, 200 µm. (F) Western blot analysis of indicated proteins in liver tissues. (G) Relative mRNA expression levels of fibrosis‐related genes in mouse livers as determined by qPCR. Data are presented as mean ± SEM. Statistical analysis was performed using Student's t‐test or multiple t‐tests. *p < 0.05, **p < 0.01, ***p < 0.001, ****p < 0.0001 (n = 3–5).

Histological analyses using H&E, Sirius Red, and F4/80 immunohistochemistry revealed that natalizumab treatment markedly reduced hepatic steatosis, fibrosis, and inflammatory cell infiltration compared with the IgG control group (Figure 8B). Moreover, natalizumab‐treated mice displayed significantly lower NAS, reduced body weight, and decreased liver‐to‐body weight ratios (Figure 8C,D). These findings prompted us to investigate whether direct ITGA4 inhibition could alter the spatial organization of liver macrophages. Strikingly, immunofluorescence analysis demonstrated that natalizumab treatment abolished the distinct zonation pattern, transforming macrophage distribution from a CV‐enriched arrangement into a disorganized, non‐zonated pattern, thereby eliminating spatial heterogeneity (Figure 8E).

Western blot analysis confirmed a significant downregulation of profibrotic and inflammatory markers, including α‐SMA, ITGA4, and phosphorylated p65 (Figure 8F). Consistent with these results, qPCR analysis revealed a significant reduction in the expression of fibrosis‐related genes (Figure 8G).

In summary, short‑term natalizumab treatment attenuated MASH phenotypes, including reduced hepatic lipid accumulation and NF‑κB activation, which may be attributed to Fc‑dependent cross‑species reactivity modulating liver macrophage activation [20, 21]. These findings support repurposing natalizumab for MASH, with a short‑term, low‑dose regimen offering translational advantages by mitigating long‑term safety concerns.

### 2.10 Endothelial Kit Regulates FSTL1 Expression via STAT2 Phosphorylation and Antibody‐Mediated Blockade of FSTL1 Reduces Macrophage Infiltration in the Liver of Kit Knockout Mice

To identify the transcription factors linking endothelial Kit to FSTL1 expression, we analyzed the FSTL1 promoter using the JASPAR database. STAT2 was predicted to bind the FSTL1 promoter with high affinity, and the top‐scoring binding site (position 1712–1726; Figure 9A, sequence shown in Figure 9B) was selected for experimental validation. A dual‐luciferase reporter assay demonstrated that co‐transfection of STAT2 with an FSTL1 promoter construct significantly reduced luciferase activity compared with the empty pcDNA3.1 vector (Figure 9C), indicating that STAT2 functions as a transcriptional repressor. Chromatin immunoprecipitation followed by qPCR using primers specific to this binding site confirmed significant enrichment of the corresponding DNA fragment relative to the IgG control, with histone H3 serving as a positive control (Figure 9D). Primer details for the predicted STAT2‐binding site are provided in Figure 9E.

**FIGURE 9:**
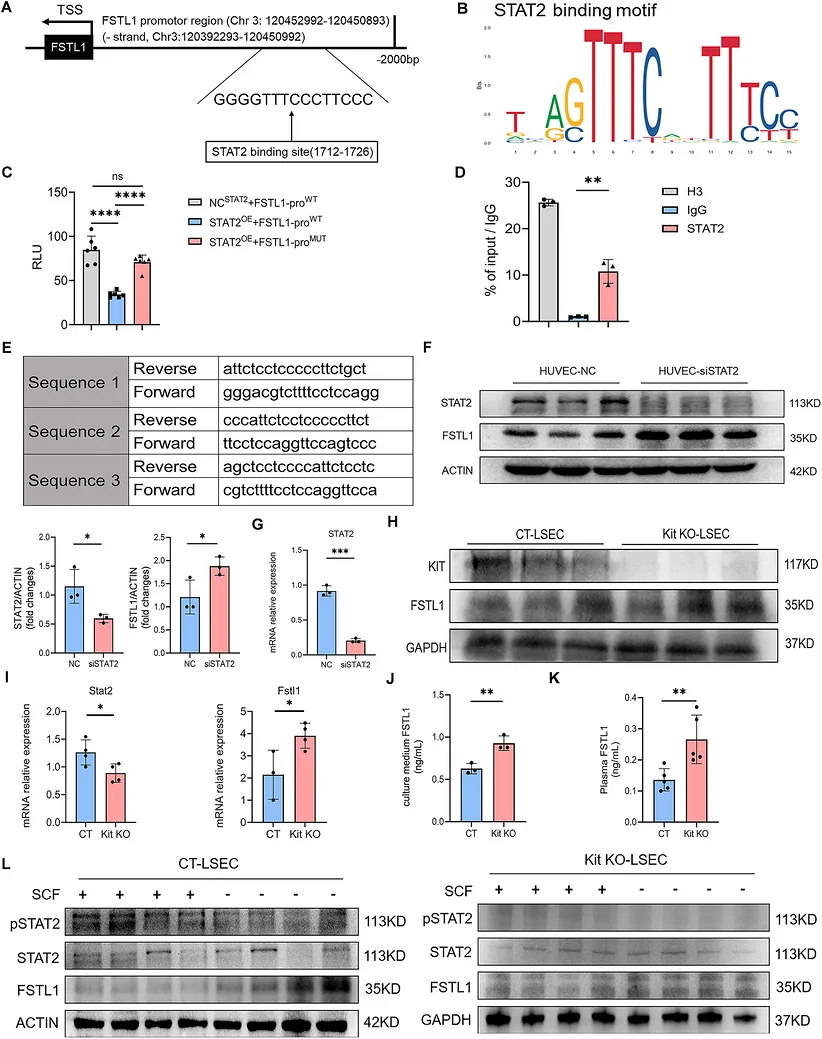
Kit signaling regulates STAT2 phosphorylation and FSTL1 expression in LSECs. (A) Schematic diagram of transcription factor STAT2 binding to the FSTL1 promoter region. (B) Predicted STAT2 binding sites within the FSTL1 promoter, as analyzed using the JASPAR database. (C) Luciferase reporter assays showing reduced luciferase activity in FSTL1 promoter fragments bound by STAT2 compared to controls. (D) qPCR analysis of chromatin immunoprecipitation (ChIP) products demonstrating STAT2 enrichment at the FSTL1 promoter region. (E) Primer sequences used for ChIP‐qPCR experiments. (F and G) Western blot analysis (F) and qPCR analysis (G) of STAT2 expression in HUVEC cells following STAT2 knockdown. (H) Western blot analysis of FSTL1 protein expression in primary LSECs from endothelial Kit KO mice and controls under basal conditions. (I) qPCR analysis of STAT2 and FSTL1 mRNA levels in primary LSECs from endothelial Kit KO and control mice. (J and K) ELISA detection of FSTL1 levels in culture medium from primary LSEC cultures (J) and in mouse serum (K) from endothelial Kit KO and control groups. (L) Immunoblot analysis of phosphorylated STAT2 (pSTAT2), total STAT2, and FSTL1 in cell lysates from the control and Kit KO LESCs. Data are presented as mean ± SEM. Statistical analysis was performed using Student's t‐test or multiple t‐tests. *p < 0.05, **p < 0.01, ***p < 0.001, ****p < 0.0001 (n = 3–5).

To functionally validate this regulatory mechanism, STAT2 was silenced in HUVECs using small interfering RNA. Both immunoblot and qPCR analyses confirmed efficient STAT2 knockdown and showed a corresponding increase in FSTL1 protein and mRNA expression (Figure 9F,G), demonstrating that STAT2 represses FSTL1 transcription.

To establish the mechanistic link with endothelial Kit, primary LSECs were isolated from chow‐fed endothelial‐specific Kit KO mice and control littermates. LSECs from Kit KO mice exhibited elevated FSTL1 expression at both the protein and mRNA levels (Figure 9H,I). ELISA measurements further confirmed increased FSTL1 secretion into the culture medium of Kit KO LSECs and elevated serum FSTL1 concentrations in Kit KO mice (Figure 9J,K). To further investigate the specific mechanism by which Kit regulates STAT2, we isolated primary liver sinusoidal endothelial cells from Kit knockout and control wild‐type mice, cultured them in vitro, and stimulated them with Stem Cell Factor (SCF), the specific ligand for Kit, to examine STAT2 phosphorylation levels and FSTL1 expression. Our results demonstrated that in control wild‐type cells, SCF stimulation significantly enhanced STAT2 phosphorylation accompanied by a marked decrease in FSTL1 expression; however, in Kit knockout cells, SCF‐induced STAT2 phosphorylation was largely abrogated and the suppressive effect on FSTL1 expression was reversed (Figure 9L). These new findings reveal that Kit functions as an upstream regulator of STAT2 by responding to SCF ligand stimulation to promote STAT2 phosphorylation, and that activated STAT2 then binds to the FSTL1 promoter to repress its transcription.

To directly establish that Kit regulates hepatic macrophage compartmentalization through FSTL1, we administered a polyclonal anti‐FSTL1 antibody to Kit KO mice on a normal diet (Figure S10A). The treatment effectively neutralized circulating FSTL1, as confirmed by serum analysis (Figure S10B). Subsequent histological examination, including H&E staining, F4/80 immunohistochemistry, and compartment‐specific macrophage immunofluorescence, revealed that FSTL1 blockade partially restored the hepatic macrophage landscape in Kit KO mice (Figure S10C). Furthermore, qPCR analysis of whole liver tissue verified successful target engagement, showing a significant reduction in FSTL1 expression, which correlated with a marked decrease in the F4/80^+^ macrophage population (Figure S10D,E).

Collectively, these findings demonstrate that endothelial Kit promotes STAT2‑mediated transcriptional repression of FSTL1, thereby maintaining endothelial homeostasis under physiological conditions.

### 2.11 FSTL1 Expression Is Elevated in Human Liver Fibrosis and Positively Correlates With Disease Severity

To assess the clinical relevance of FSTL1 in human liver disease, we first analyzed public datasets for hazard ratios associated with FSTL1 expression across multiple organ systems [22]. When filtered for liver‐related conditions, elevated FSTL1 levels were found to significantly increase the risk of liver fibrosis and other hepatic disorders (Figure S11A). Consistently, analysis of gene expression profiles from cirrhotic livers in the dataset reported by Moylan et al. identified FSTL1 as one of the genes upregulated across progressive stages of cirrhosis, with its expression positively correlating with disease severity (Figure S11B) [23].

Further examination of the GSE260666 patient cohort revealed that endothelial adhesion‐related molecules, including LAMC1 and VCAM1, as well as the macrophage integrin receptor ITGA4, were significantly upregulated in MASH patients compared with healthy controls (Figure S11C).

Spearman correlation analysis (GSE193084) showed that FSTL1 expression positively correlated with ITGA4, VCAM1, and ICAM1, but inversely correlated with STAT2 (Figure S11D). Subsequently, we quantified the protein levels of FSTL1 and ITGA4 in liver samples from patients in our center, and the results were consistent with the bioinformatics analysis (Figure S11E).

## 3 Discussion

As highly specialized vascular components, LSECs constitute a critical interface in hepatic physiology, maintaining tissue homeostasis through their unique structural and functional characteristics. Their fenestrations facilitate dynamic exchanges with circulating cells and plasma constituents, thereby enabling intricate communication with diverse hepatic cell populations, including immune cells such as macrophages [2, 24, 25, 26, 27]. Within this complex multicellular network, Kit (CD117)‐a type III receptor tyrosine kinase‐serves as a specific marker of pericentral LSECs and provides a valuable tool for identifying and investigating this specialized endothelial subset [28, 29].

The functional repertoire of LSECs is further expanded by their expression of FSTL1, a multifunctional secreted glycoprotein that circulates in the bloodstream and exhibits context‐dependent biological activities. Initially identified as a myokine modulating interferon regulatory factor 4‐mediated apoptotic signaling [30], FSTL1 has since been detected in several organs, including the liver and central nervous system [9]. Of particular relevance to hepatic pathology, FSTL1 expression is elevated in mesenchymal stem cells, endothelial cells, and macrophages during fibrotic liver remodeling, and circulating FSTL1 levels have been proposed as a potential diagnostic biomarker for advanced fibrosis [10, 31]. Despite these insights, the specific functions of LSEC‐derived FSTL1 have remained unclear. Our integrative analysis of single‐cell and bulk RNA‐sequencing datasets revealed a pronounced upregulation of endothelial FSTL1 expression in MASH, accompanied by distinctive zonal heterogeneity across the hepatic lobule. Most notably, we identified a previously uncharacterized role for LSEC‐derived FSTL1 in mediating macrophage interactions and determining their spatial organization within the hepatic microenvironment.

The mechanistic basis of FSTL1‐mediated signaling involves its interaction with ITGA4, a key member of the integrin α‐chain family that typically forms heterodimeric complexes with β1 or β7 subunits [32]. Unlike many ubiquitously expressed integrins, ITGA4 exhibits notable cell‐type specificity, with predominant expression in myeloid immune cells (including macrophages), lymphocytes, and hematopoietic stem cells. This restricted distribution underscores its central role in immune surveillance, inflammatory signaling, and modulation of the tumor microenvironment [33, 34, 35, 36]. Our findings demonstrate that macrophage‐expressed ITGA4 functions as the principal receptor for FSTL1, initiating downstream NF‐κB signaling cascades that drive macrophage activation and promote M1 polarization. This discovery carries particular therapeutic significance given that natalizumab‐a clinically approved monoclonal antibody targeting ITGA4‐has shown efficacy in immune‐mediated disorders such as inflammatory bowel disease [37, 38, 39, 40]. However, translation of this therapeutic strategy to MASH has been constrained by safety concerns associated with sustained ITGA4 inhibition. Among the most clinically relevant outcomes of this study is the notable efficacy of a short‐term, low‐dose natalizumab regimen. Our experimental data demonstrate that even at substantially reduced doses and treatment durations compared with standard clinical protocols, this modified approach effectively attenuates hepatic macrophage activation and ameliorates key pathological features of MASH. These results support the presence of a potential therapeutic window for MASH management‐distinct from established treatment paradigms‐that may deliver clinical benefits while minimizing the risks associated with prolonged integrin blockade.

The collective findings of this study position the LSEC Kit/FSTL1/ITGA4 signaling axis as a critical regulatory pathway linking endothelial dysfunction to macrophage‐mediated inflammation in MASH pathogenesis. Beyond elucidating fundamental disease mechanisms, our work highlights several potential therapeutic intervention points along this pathway, including FSTL1 neutralization and ITGA4 blockade. The demonstrated efficacy of natalizumab, together with its established clinical safety profile, presents a strong rationale for therapeutic repurposing in MASH‐an increasingly prevalent condition with limited effective treatment options. Future investigations should aim to validate these observations in larger human cohorts, explore potential synergistic effects between ITGA4 inhibition and emerging MASH therapies, and delineate the temporal dynamics of this signaling axis during both disease progression and regression. Such efforts may identify optimal intervention windows and guide the development of personalized therapeutic strategies for patients with metabolic dysfunction‐associated steatohepatitis.

## 4 Material and Methods

### 4.1 Animal Studies

Male C57BL/6J mice (8 weeks old) were purchased from Beijing Vital River Laboratory Animal Technology Co., Ltd. The generation of Cdh5CreERT Kitflox/flox (Kit KO) and Cdh5CreERT Fstl1flox/flox (Fstl1 KO) mice has been described previously [13], and the detailed construction information of the targeting vectors is provided in Table S1. Genotyping of transgenic offspring was performed by PCR using mouse tail DNA as the template, followed by agarose gel electrophoresis. To induce Cre recombinase activity, 6‐week‐old male mice received intraperitoneal (i.p.) injections of tamoxifen (100 mg/kg) for five consecutive days. Littermates injected with the tamoxifen vehicle served as control groups to exclude potential off‐target effects. Mice were analyzed at least one week after the final tamoxifen injection.

To establish a mouse model of MASLD with varying severity, 8‐week‐old male mice were fed a CDAA diet containing 45 kcal% fat diet for 1–10 weeks(Table S2).

For the in vivo recombinant murine FSTL1 (rmFSTL1) experiments, 8‐week‐old wild‐type C57BL/6J mice received daily i.p. injections of rmFSTL1 (0.1 mg/kg/day) for one week. Control mice were injected with an equivalent volume of PBS. In a separate group, anti‐VLA4 antibody (1 mg/kg/day) was co‐administered i.p. with rmFSTL1 for one week, while the corresponding control group received an equivalent dose of Rat IgG. Mice were euthanized 24 h after the final injection.

For therapeutic intervention in the CDAA model, 8‐week‐old wild‐type C57BL/6J mice were fed a CDAA diet for 7 weeks, followed by daily i.p. injections of anti‐VLA4 antibody (4 mg/kg/day) for one week. Control mice received an equivalent dose of Rat IgG. Mice were euthanized 24 h after the last dose.

For natalizumab treatment in the CDAA model, 8‐week‐old wild‐type C57BL/6J mice were fed a CDAA diet for 7 weeks, followed by NTZ administration (5 mg/kg) via tail vein injection every other day for one week. Control mice received an equivalent dose of Human IgG. Mice were euthanized 24 h after the final administration.

For the in vivo neutralization of FSTL1 in Kit KO mice, 6‐week‐old mice were intraperitoneally administered tamoxifen for five consecutive days. After a one‐week interval, the mice received intraperitoneal injections of pAbFSTL1 (abclonal, A15789) at a dose of 20 µg per mouse per day, administered every other day for a total of three injections. Control mice were given an equivalent dose of PBS. Tissues were collected for analysis 24 h after the final injection.

All animals were maintained under specific pathogen‐free conditions with a 12‐h light/dark cycle and had ad libitum access to food and water. All experimental procedures were conducted in compliance with the National Academies' Guide for the Care and Use of Laboratory Animals and were approved by the Animal Ethics Committee of Air Force Medical University (Xi'an, China) under protocol number KY20253406‐1.

### 4.2 Metabolic Cage Analysis

All mice were individually housed in a comprehensive lab animal monitoring system (PhenoMaster, TSE) equipped with photoelectric cells, and fed a CDAA diet. Following a 12‐h acclimatization period, metabolic data were collected continuously for 48 h, with measurements taken every 40 min. Energy expenditure (EE) was calculated from the oxygen consumption (VO_2_) and carbon dioxide production (VCO_2_) values. Data were compared between groups based on the average values for each light (inactive) and dark (active) phase.

### 4.3 Cell Culture and in Vitro Experiments

The THP‐1 cell line was purchased from Fenghui Biology (Hunan, China), while the HUVEC and HEK 293T cell lines were obtained from the Cell Bank of the Chinese Academy of Sciences (Shanghai, China). THP‐1 cells were cultured in a specialized THP‐1 medium (Procell), and HUVEC and HEK 293T cells were maintained in DMEM supplemented with 10% FBS and 1% penicillin/streptomycin.

#### 4.3.1 rhFSTL1 Treatment of Macrophages

THP‐1 cells were seeded in 6 or 12 well plates at 70%–80% confluence. The cells were first treated with 100 ng/mL PMA for 48 h to induce adherence. The medium was then replaced, and the cells were treated with different concentrations of rhFSTL1 for another 48 h before being harvested for subsequent analysis.

#### 4.3.2 ITGA4 Inhibitor Assay

THP‐1 cells were treated with PMA for 48 h, followed by incubation with the ITGA4 inhibitor BIO‐1211 (4 nmol/mL) for 4 h. Subsequently, rhFSTL1 (200 ng/mL) was added to the culture for an additional 48 h.

#### 4.3.3 Genetic Manipulation for Overexpression

For FSTL1 overexpression, the amplified FSTL1 fragment was cloned into the pHBLV‐CMV‐MCS‐3Flag‐ZsGreen‐Puro lentiviral vector (Hanbio Co., Ltd.) and transfected into HUVEC cells. Stably transduced cells were selected and maintained in culture medium containing puromycin.

#### 4.3.4 Migration Assay

FSTL1‐overexpressing HUVEC cells were plated in the lower chamber of a 24‐well plate, while THP‐1 cells were seeded in the upper chamber of a transwell insert. After 24 h of co‐culture, the THP‐1 cells that had migrated were collected for further analysis.

#### 4.3.5 FSTL1 Blocking in Co‐Culture

Conditioned medium collected from FSTL1‐overexpressing HUVEC cultures was placed in the lower chamber. THP‐1 cells were seeded in the upper chamber. In the experimental group, an FSTL1 polyclonal antibody (4 µg/mL) was added to the medium, whereas the control group received an equivalent dose of rabbit‐derived IgG. After 72 h of treatment, cells were harvested for subsequent analysis.

#### 4.3.6 SCF Stimulation Experiment on LSECs

LSECs were isolated from Kit KO and control mice, seeded into 6‐well plates at a density of 2 × 106 cells per well, and cultured in ECM medium for 24 h. After medium replacement, the cells were treated with SCF protein (2 µg/mL) or an equal volume of PBS for 48 h, followed by protein extraction for subsequent experiments. Detailed information of all reagents is provided in Table S3.

### 4.4 Histology

Liver tissues were fixed with 4% paraformaldehyde for 3 h. Hematoxylin and eosin (H&E) staining, Sirius Red staining, immunofluorescence (IF), and immunohistochemistry were carried out following a previously described protocol. Images were then processed using CaseViewer.

### 4.5 Quantitative Real‐Time PCR

Total RNA was extracted from cells or tissues using Trizol reagent and reverse‐transcribed into cDNA with a commercial reverse transcription kit. Quantitative real‐time PCR (qPCR) was performed using a SYBR Green PCR master mix. The expression levels of all the targeted genes were normalized to those of β‑actin, and the fold changes were calculated via the 2^−ΔΔCt^ comparative method. All primer sequences are listed in Table S4.

### 4.6 Western Blotting

Total protein was extracted using RIPA lysis buffer supplemented with protease inhibitors, and the protein concentration was determined using a BCA protein assay kit. Equal amounts of protein were separated by SDS‐polyacrylamide gel electrophoresis (SDS‐PAGE) and transferred onto polyvinylidene difluoride (PVDF) membranes. The membranes were blocked with 5% skim milk and subsequently incubated with appropriately diluted primary and secondary antibodies (Table S5). Protein signals were finally detected using a ChemiDoc MP Imaging System (Bio‐Rad).

### 4.7 Flow Cytometry

Mouse NPCs were isolated as previously described and stained with anti‑mouse CD45‑PerCP (103129, BioLegend), anti‑mouse F4/80‐PE (111603, BioLegend), CD11b‑Pacific Blue (101223, BioLegend), and Ly6C‐FITC (128005, BioLegend). Viability was assessed using 7‑AAD (420404, BioLegend). Flow cytometry was performed on a Sony MA900, and data were analyzed with Cell Sorter software (Sony). A total of 50,000 events were acquired. The gating strategy was: (i) main cell population (FSC‑A vs. SSC‑A); (ii) singlet discrimination (FSC‑H vs. FSC‑A); (iii) live cell gate (7‑AAD); (iv) macrophages (CD11b^+^F4/80^+^) within the CD45^+^ gate; (v) pro‑inflammatory subset defined as Ly6Chigh within the macrophage gate.

THP‐1 cells were cultured and stimulated as required. Cells were then harvested, stained with viability dye (7‑AAD, 420404, BioLegend), followed by fixation and permeabilization using a commercial intracellular staining buffer set. Cells were subsequently stained with anti‑human TNF‑α‑PerCP (502923, BioLegend) and anti‑human IL‑1β‑Alexa Fluor 647 (511707, BioLegend).

### 4.8 Biochemical Analysis

Serum levels of alanine aminotransferase (ALT) and aspartate aminotransferase (AST) were measured using commercial assay kits according to the manufacturer's instructions on a Chemray240 automatic biochemistry analyzer (Rayto). Detailed information is listed in Table S6.

### 4.9 Luciferase Reporter Assay

The pCMV‐MCS‐3*Flag‐STAT2 expression vector and the firefly luciferase reporter vector containing the FSTL1 promoter (pGL3‐basic‐FSTL1) were constructed by Paiwei Biotechnology. HEK 293T cells were seeded in 96‐well plates. After overnight culture, the cells were co‐transfected using Lipofectamine 2000 according to the manufacturer's protocol. The transfection mixtures included either the empty pCMV‐MCS vector or different concentrations of the pCMV‐MCS‐3*Flag‐STAT2 expression vector, together with the pGL3‐basic control plasmid, pGL3‐basic‐FSTL1‐pro‐WT, or pGL3‐basic‐FSTL1‐pro‐mut plasmids. A Renilla luciferase plasmid was co‐transfected as an internal control. Reporter (luciferase) activity was measured using a Dual‐Luciferase Reporter Assay Kit (Beyotime Biotechnology).

### 4.10 ELISA

The expression levels of secreted FSTL1 were measured using a human FSTL1 ELISA kit or a mouse Fstl1 ELISA kit (Jianglai Biotechnology), according to the manufacturer's instructions. In brief, standards or samples were added to the pre‐coated wells and incubated at room temperature with gentle shaking for 2.5 h. Subsequently, biotinylated antibody and streptavidin solution were added, followed by a one‐step TMB substrate for color development. The absorbance was measured at 450 nm using an ELISA microplate reader to determine FSTL1 protein levels. Detailed information is listed in Table S6.

### 4.11 Human Samples

Liver tissues were obtained from patients with biopsy‐proven MAFLD who underwent a liver biopsy during bariatric surgery at Xijing Hospital and non‐MAFLD (control) patients who were diagnosed with liver haemangioma and accepted liver surgeries. All procedures were approved by the Human Ethics Committees of Xijing Hospital under approval number KY20260215. All participants provided informed written consent.

### 4.12 Bulk RNA Sequencing

Total RNA was extracted using Trizol reagent from the following samples: Kit± liver sinusoidal endothelial cells (LSECs) and primary macrophages isolated from the livers of male mice on a standard chow diet, as well as RAW264.7 cells treated with 100 ng/mL recombinant protein for 24 h. The extracted mRNA was enriched using mRNA capture beads. Following bead purification, the mRNA was fragmented under elevated temperature. The fragmented mRNA was then used as a template to synthesize first‐strand cDNA in a reverse transcriptase master mix. Second‐strand cDNA synthesis was performed simultaneously with end repair and poly(A)‐tailing. Adapter ligation was subsequently carried out, and target fragments were selected and purified using Hieff NGS DNA Selection Beads. The library was then amplified by PCR, and final sequencing was performed on an Illumina NovaSeq 6000 system.

### 4.13 Bioinformatic Analysis

Bioinformatic analysis of the bulk RNA sequencing data was conducted using the Omicsmart platform (https://www.omicsmart.com/). The data presented in Figures S1C, S6C, and S3A were sourced from the dataset GSE248077. Data for Figures S2B and S3C were obtained from GSE213165 and GSE140994, respectively. Data for Figure S7A,B were derived from GSE166504, while data for Figure S11D,E originated from GSE260666 and GSE193084.

### 4.14 Statistical Analysis

Statistical analyses were performed using GraphPad Prism software (version 10). Data are presented as mean ± error of the mean (SEM). All datasets were tested for normality and homogeneity of variance. For comparisons between two groups, the Student's t‐test was applied for normally distributed data, and the Mann–Whitney U test was used for non‐normally distributed data. The Spearman's rank correlation coefficient was calculated for nonnormally distributed data. A *p*‐value of less than 0.05 was considered statistically significant. The following symbols are used to indicate statistical significance throughout the figures: *p < 0.05; **p < 0.01; ***p < 0.001; ****p < 0.0001; ns, not significant.

## Author Contributions

Conceptualization, L.W., J.L.D., and F.H.; clinical screening, P.S. and J.J.L.; formal analysis, L.W., J.L.D., and F.H.; investigation, Y.W.L., W.D., and H.X.; resources, L.S., Z.S.Y., and Z.Q.F.; data curation, L.S., Z.S.Y., and Z.Q.F; writing – original draft, L.S., Z.S.Y., and Z.Q.F; writing – review and editing, all authors and visualization, L.W., J.L.D. and F.H.; supervision, L.W., J.L.D. and F.H.; funding acquisition, L.W. and Z.S.Y.

## Conflicts of Interest

The authors declare no conflicts of interest.

## Supporting information




**Supporting File**: advs76410‐sup‐0001‐SuppMat.docx.

## Data Availability

The raw sequencing data generated in this study have been deposited in the NCBI Sequence Read Archive (SRA) and GEO under accession numbers PRJNA1426135 (RNA‐seq data of primary liver macrophages from Kit KO and control mice fed a normal chow diet), GSE319522 (RNA‐seq data of RAW264.7 cells treated with recombinant FSTL1 protein or PBS), and GSE216426(RNA‐seq data of Kit‐ LSECs and Kit+ LSECs).
