# Supplementary material for: Liver Endothelia Orchestrate MASH‐Associated Macrophage Zonation Through FSTL1‐ITGA4 Axis
Source: Adv Sci (Weinh). 2026 Aug 3:e76410. Online ahead of print. doi: 10.1002/advs.76410 (PMC13431240; doi:10.1002/advs.76410)
Supplement: Supplementary file 1 — Supporting File: advs76410‐sup‐0001‐SuppMat.docx. [file ADVS-9999-e76410-s001.docx]

**Liver Endothelia Orchestrate MASH-associated Macrophage Zonation through FSTL1-ITGA4 Axis**

Lin Sun, Zhensheng Yue, Zhiqiang Fang, Hao Xu, Wei Du, Yuwei Ling, Jingjing Liu, Ping Song, Fei He, Juanli Duan, Lin Wang

**Supplementary figures**


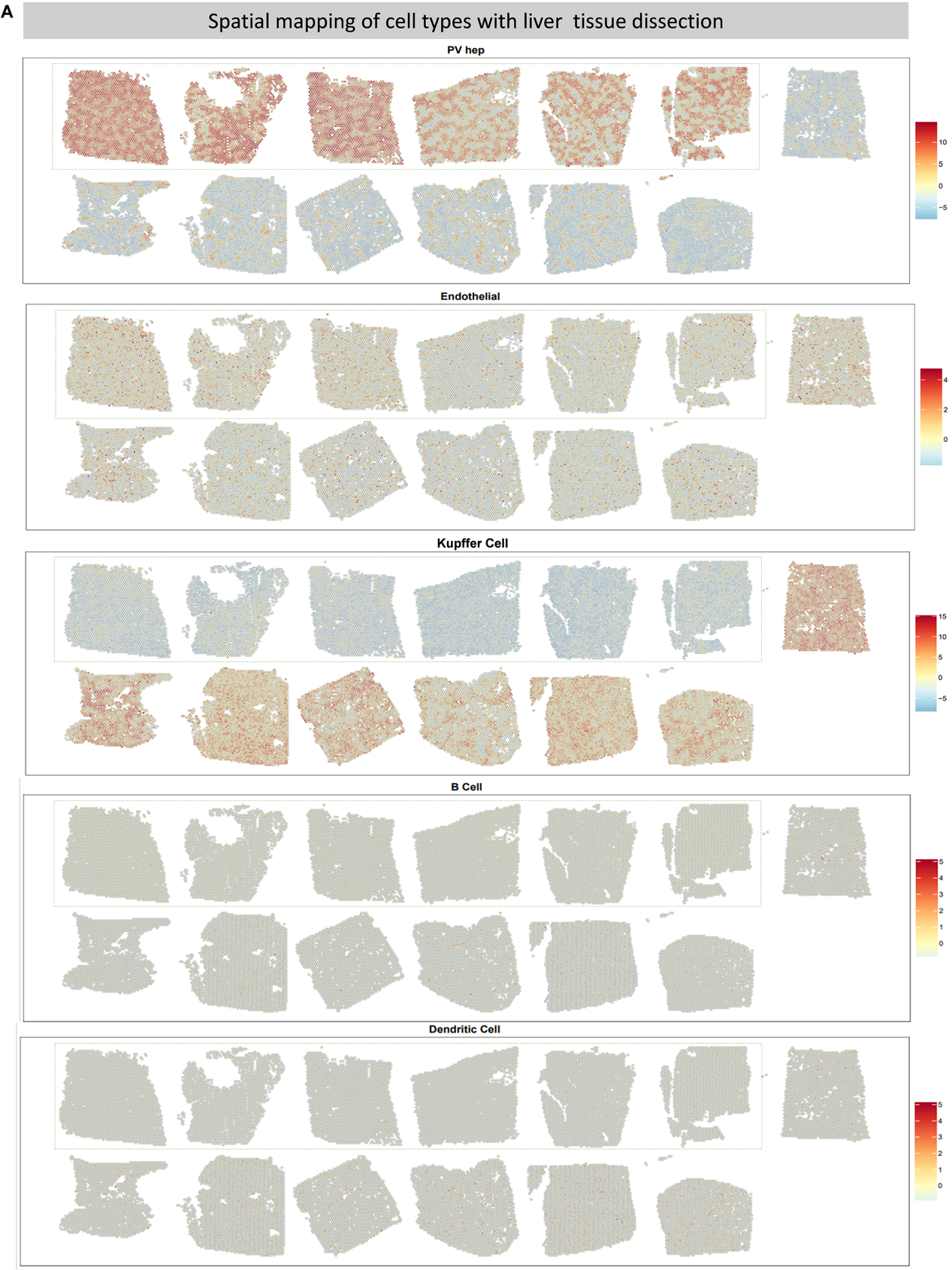


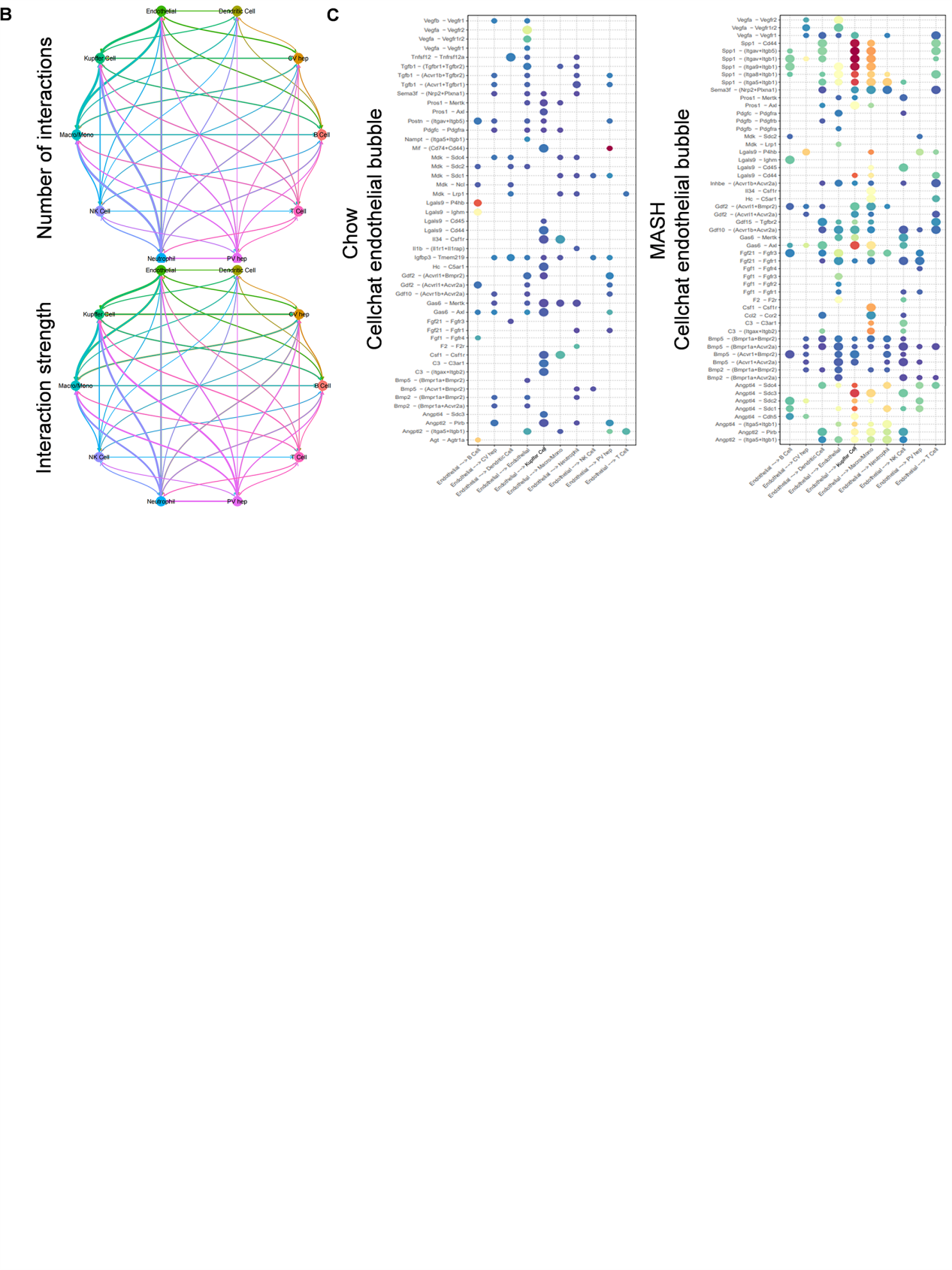


**Figure. S1. Spatial heterogeneity of hepatic macrophages in MASH mice**

(A) Spatial transcriptomics and scRNA‑seq integration of mouse liver. Shown are periportal hepatocytes (PV hep; Cyp2f2, Hal), endothelial cells (Pecam1, Cdh5, Lyve1, Kdr, Oit3), Kupffer cells (C1qa, C1qc, Csf1r, Clec4f), B cells (Ebf1, Cd79a, Cd79b, Ms4a1), and dendritic cells (Lsp1, H2afy, Siglech, Runx2, Ccr9). Color intensity indicates marker gene expression level per spot.

(B) Network diagram depicting cell-cell interactions among the identified cell clusters.

(C) Dot plot comparing the interactions between endothelial cells and other cell types in normal diet and MASH mice.


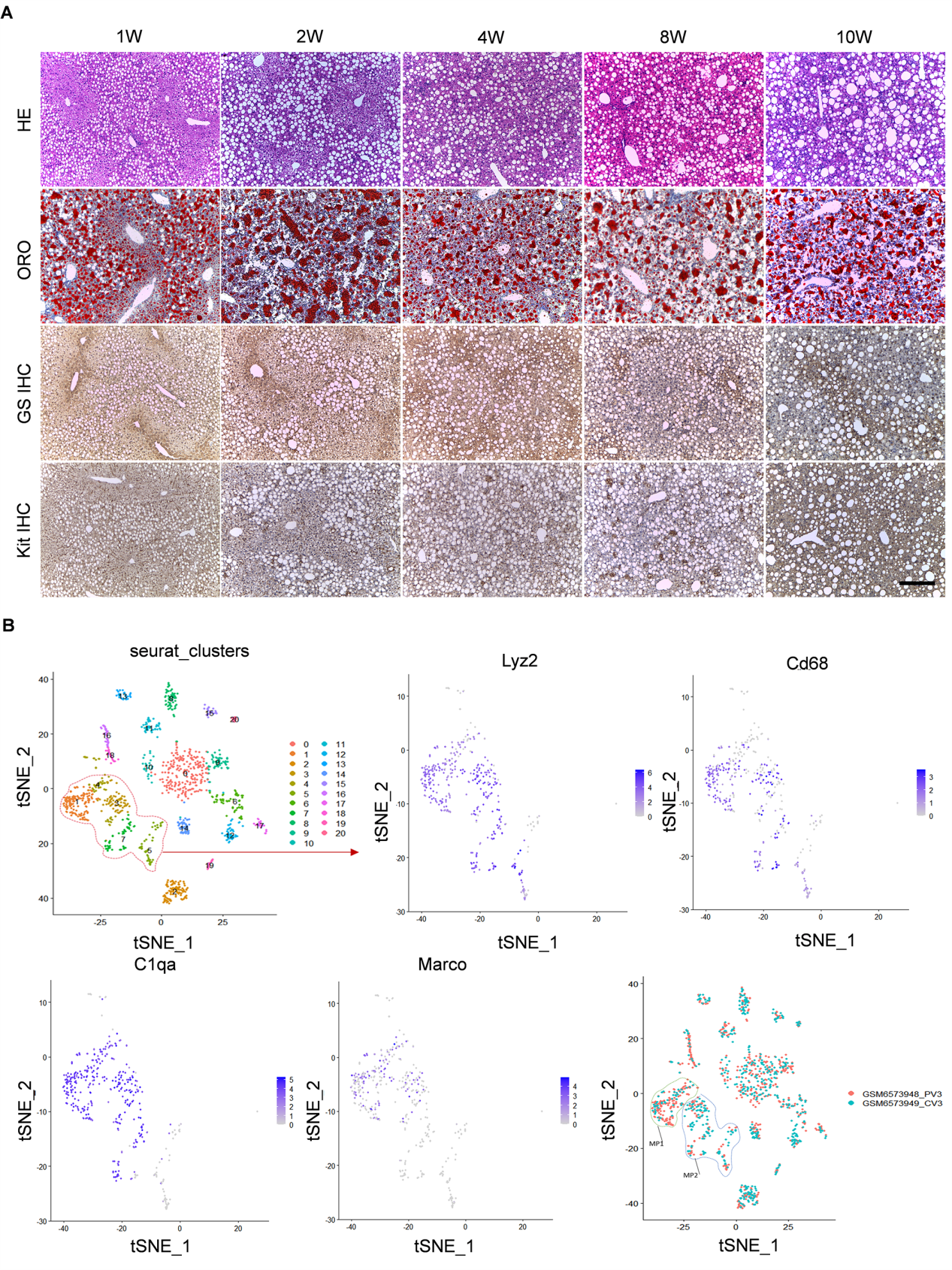


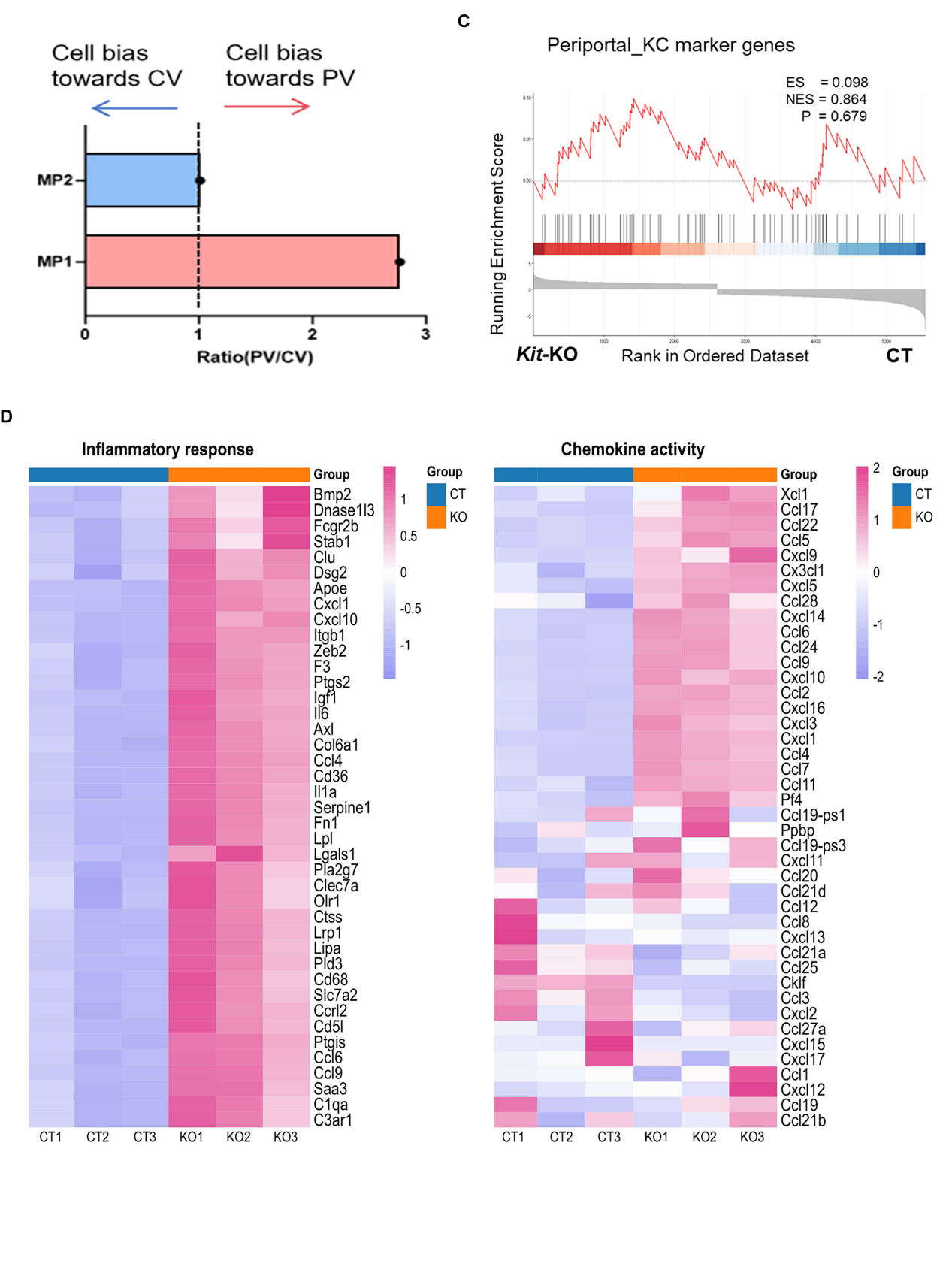


**Figure. S2. CDAA diet alters macrophage zonation, and endothelial Kit knockout affects macrophage distribution.**

(A) Representative images of H&E staining, Oil Red O staining, glutamine synthetase (GS) immunohistochemistry, and Kit immunohistochemistry in liver tissues from mice fed a CDAA diet for different durations. Scale bars; 200 µm.

(B) Re-analysis of zonal macrophage distribution using dataset GSE213165.

(C) GSEA comparing primary macrophages from Kit KO and control mice with PV zone macrophages.

(D) Heatmap showing differentially expressed genes related to inflammation and chemokines in primary macrophages from endothelial Kit KO versus control mice.


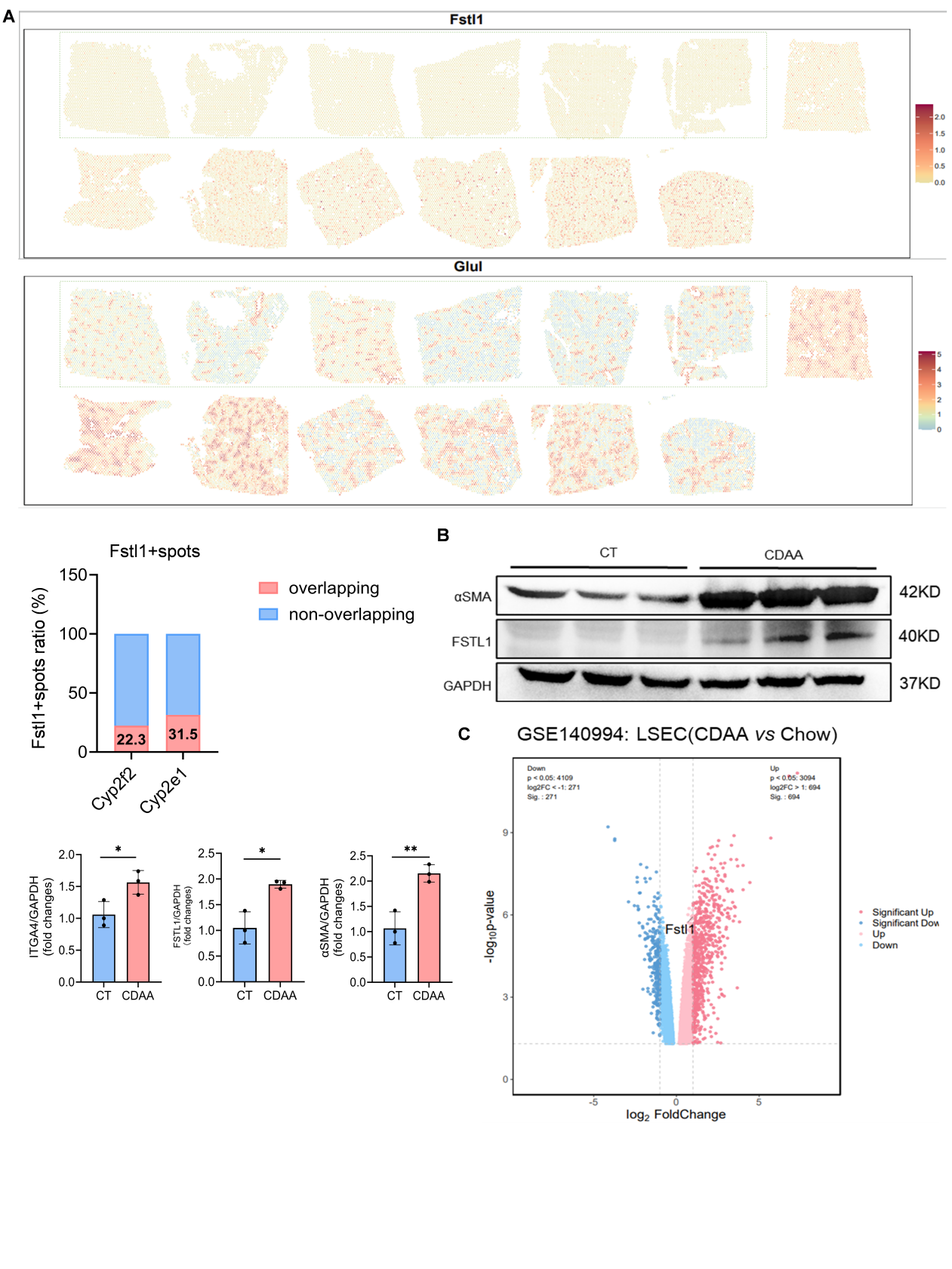


**Figure. S3. FSTL1 expression is upregulated in MASH.**

(A) Re-analysis of Fstl1 and Glul expression from a spatial transcriptome dataset of normal mouse liver (GSE248077). The bar graph shows the proportion of Fstl1-positive spots that co-localize with Glul-positive spots.

(B) Immunoblot analysis of the indicated proteins in liver lysates from mice fed a CDAA or control diet for 10 weeks.

(C) Re-analysis of the GEO dataset (GSE140994) showing a volcano plot of differentially expressed genes in endothelial cells from CDAA-fed versus control mice. Fstl1 is highlighted. Data are presented as mean ± SEM (n = 3). Statistical significance was determined using Student's t-test or multiple t-tests (*p < 0.05, **p <0.01).


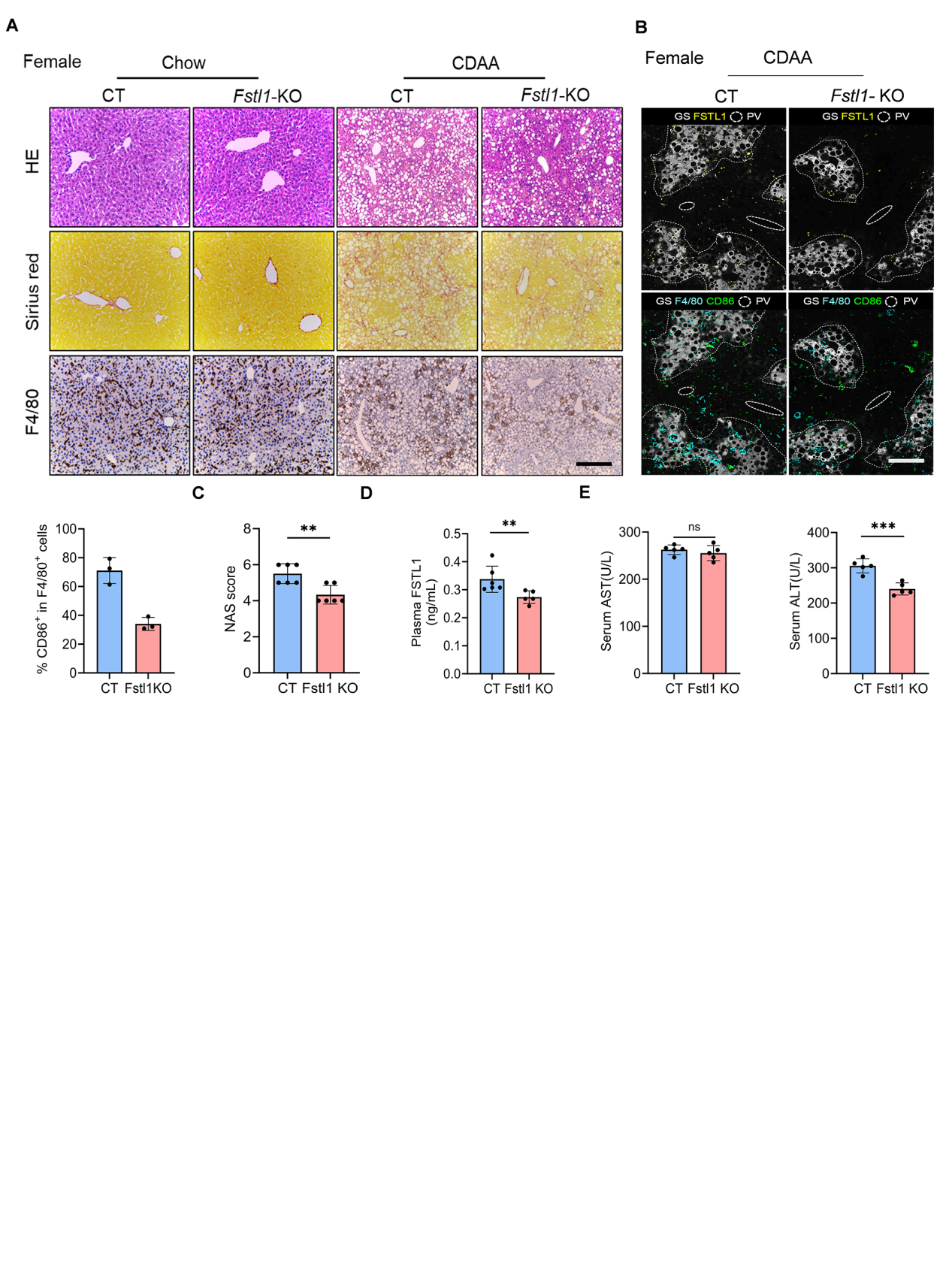


**Figure. S4. Increased perivenous macrophages are alleviated by endothelial FSTL1 knockout in a sex-independent manner.**

(A) Representative H&E staining, Sirius Red staining, and F4/80 immunohistochemistry in liver sections from female mice fed either a control diet (scale bar; 200 µm) or a CDAA diet (scale bar; 100 µm).

(B) Representative immunofluorescence images and quantification of F4/80, CD86, and GS in liver sections from the two groups of female mice. Scale bars; 200 µm.

(C and D) NAS and serum FSTL1 levels in the two groups of mice.

(E) Serum AST and ALT levels in two groups of mice. Data are presented as mean ± SEM (n = 3-6). Statistical significance was determined by two-sample t-test (*p < 0.05, **p < 0.01, ***p < 0.001; ns, not significant).


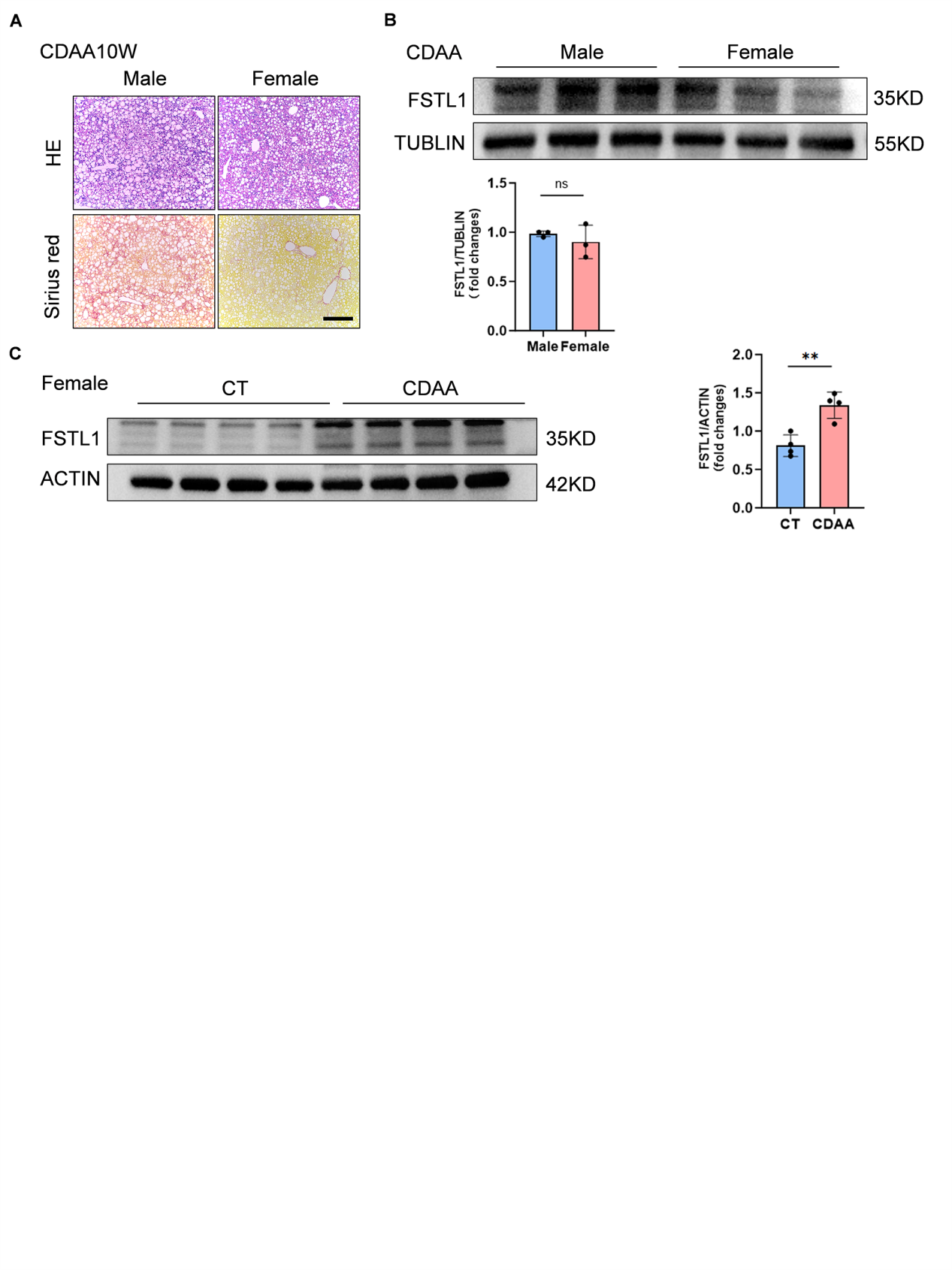


**Figure. S5. Sex differences in MASH phenotype and hepatic FSTL1 expression in mice fed a CDAA diet.**

(A) Representative images of liver sections stained with H&E and Sirius Red from male and female mice fed a CDAA diet for 10 weeks. Scale bars; 100 µm.

(B) Immunoblot analysis of FSTL1 protein expression in the livers of male and female mice fed a CDAA diet.

(C) Immunoblot analysis comparing hepatic FSTL1 expression in female mice fed a normal diet or a CDAA diet. Data are presented as mean ± SEM (n = 3-4). Statistical significance was determined by two-sample t-test (**p < 0.01, ns, not significant).


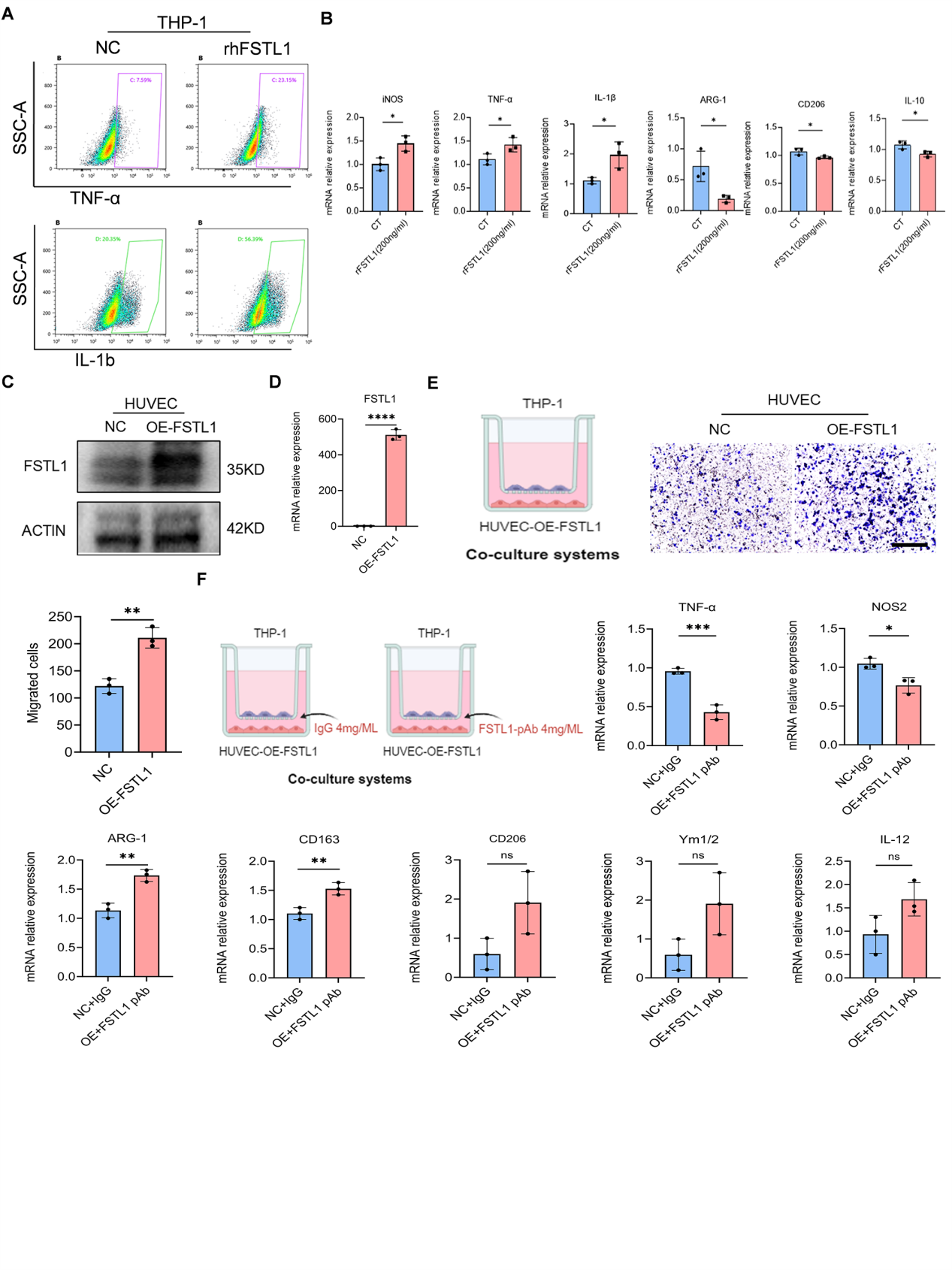


**Figure. S6. FSTL1 promotes macrophage M1 polarization and migration.**

(A) Gating strategy for flow cytometry and representative flow cytometry plots.

(B) Expression of relevant inflammatory factors in THP-1 cells treated with or without FSTL1, as determined by qPCR.

(C and D) FSTL1 protein (C) and mRNA (D) levels in HUVECs overexpressing FSTL1.

(E) Transwell migration assay of THP-1 cells co-cultured with OE-FSTL1-HUVEC or NC-Vector-HUVEC.

(F) qPCR analysis of inflammatory molecules in THP-1 cells co-cultured with OE-FSTL1-HUVEC or NC-Vector-HUVEC in the presence of an FSTL1 polyclonal antibody. Data are presented as mean ± SEM (n = 3). Statistical analysis was performed using Student's t-test or multiple t-tests (*p < 0.05, **p < 0.01, ***p < 0.001, ****p < 0.0001).


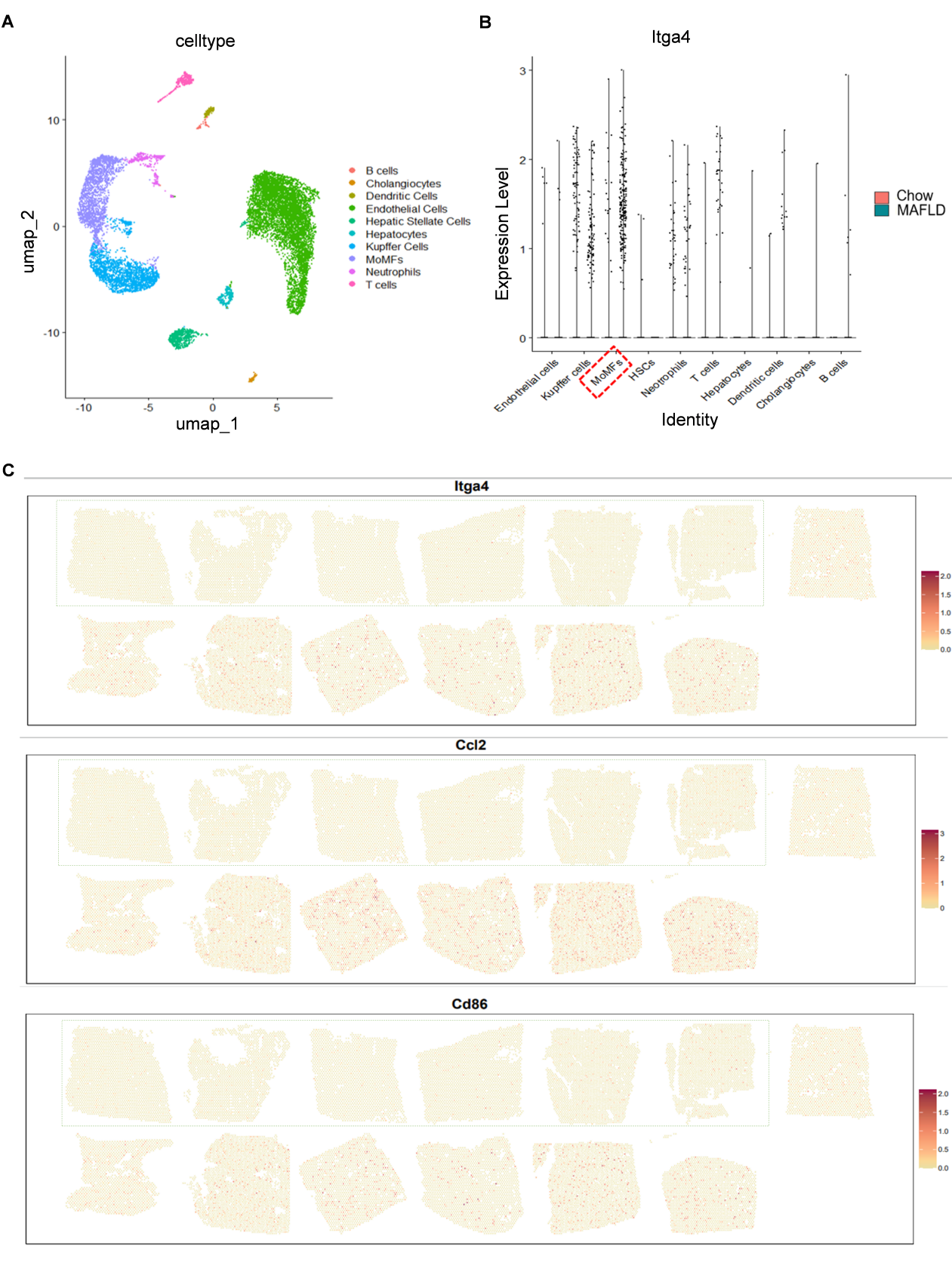


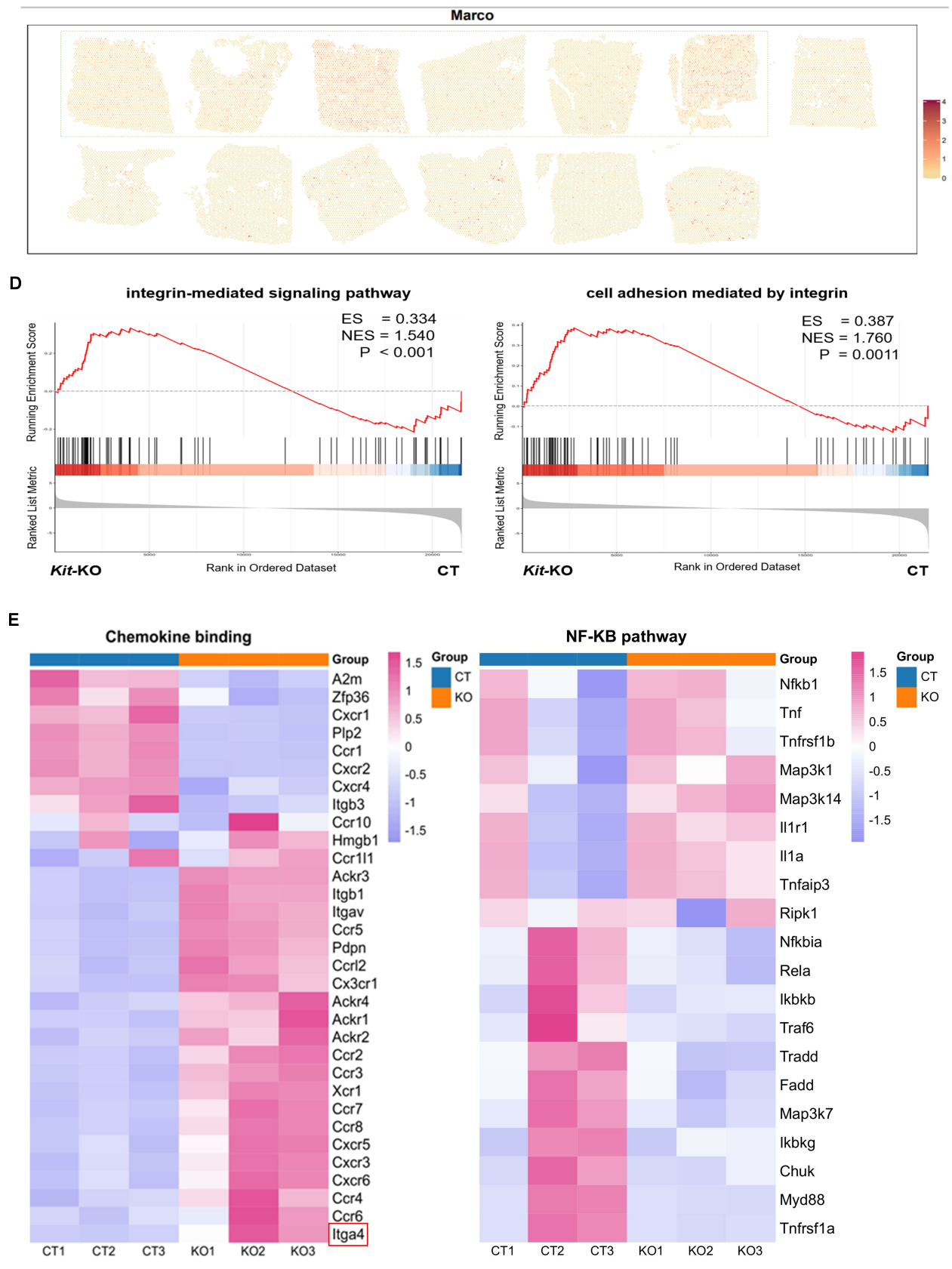


**Figure. S7. FSTL1 regulates macrophage M1 polarization and migration via interaction with integrin α4.**

(A and B) Analysis of a public dataset (GSE166504) shows differential expression of ITGA4 in macrophages between the two indicated groups. Left: Chow; Right: MAFLD .

(C) The expression levels of Itga4, Ccl2, Cd86, and Marco in GSE248077.

(D) GSEA of integrin-related signaling pathways in primary macrophages from endothelial Kit KO versus control mice.

(E) Heatmap displaying differentially expressed genes associated with chemokine and NF-κB pathways in primary macrophages from endothelial Kit KO and control mice.


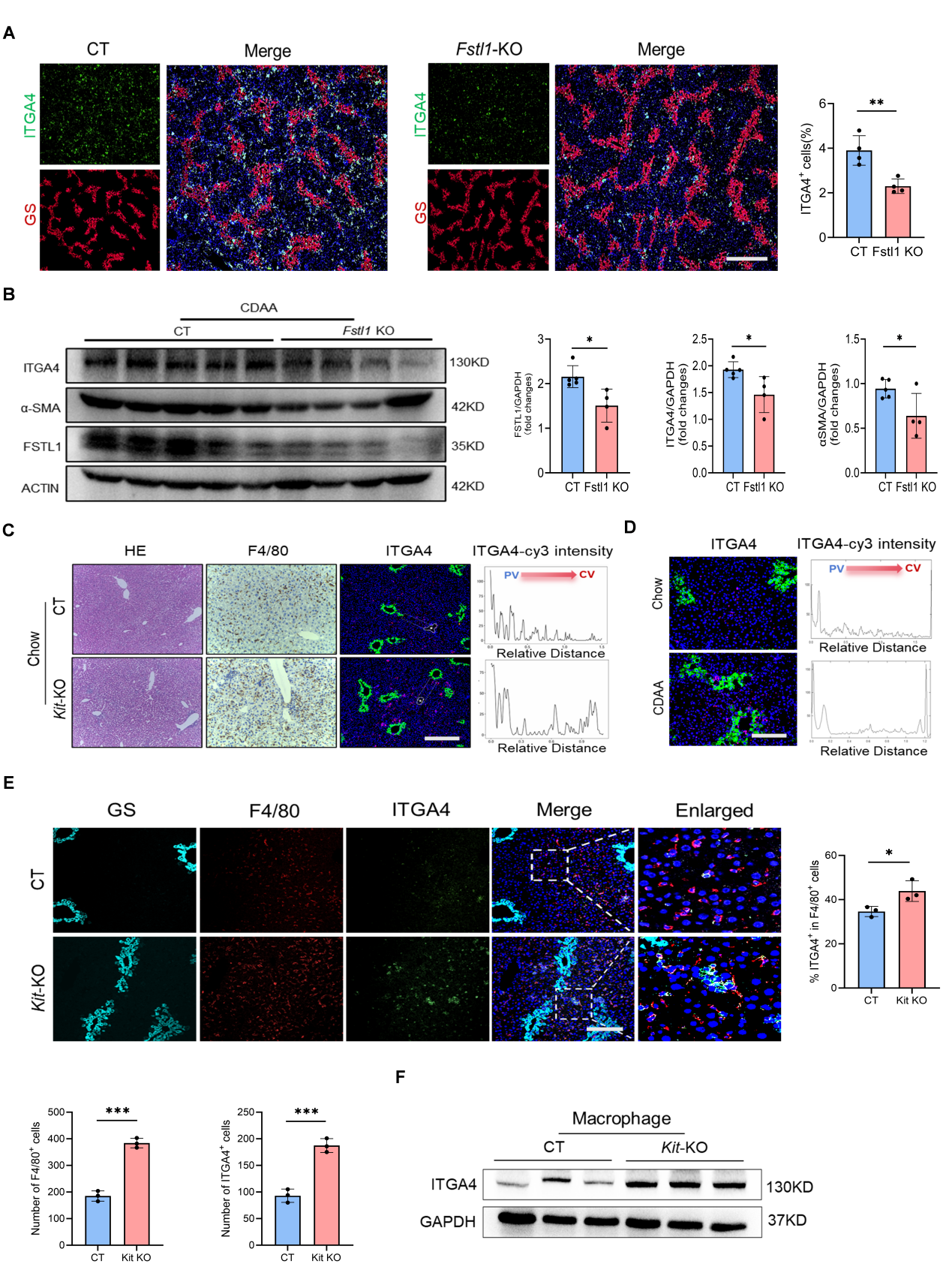


**Figure. S8. Endothelial FSTL1 deletion attenuates MASH-induced enrichment of ITGA4-expressing macrophages in the central vein region**

(A) Representative immunofluorescence images of ITGA4 (red) and GS (green) in liver sections from FSTL1 KO and control mice fed a CDAA diet. Scale bars; 200 µm.

(B) Immunoblot analysis of indicated proteins in liver lysates from FSTL1 KO and control mice under CDAA feeding.

(C) Representative H&E staining, F4/80 immunohistochemistry, and immunofluorescence of ITGA4 (red) and GS (green) in liver sections from endothelial Kit KO and control mice. Scale bars; 200 µm.

(D) Representative immunofluorescence images of ITGA4 (red) and GS (green) in liver sections from CDAA-fed and control mice. Scale bars; 200 µm.

(E) Immunofluorescence staining of F4/80, ITGA4, and GS in liver sections from endothelial Kit KO and control mice. Scale bars; 200 µm.

(F) Immunoblot analysis showing ITGA4 expression in primary macrophages from endothelial Kit KO versus control mice. Data are presented as mean ± SEM (n = 3–5). Statistical significance was determined by Student’s t-test or multiple t-tests (*p < 0.05, **p < 0.01, ***p < 0.001).


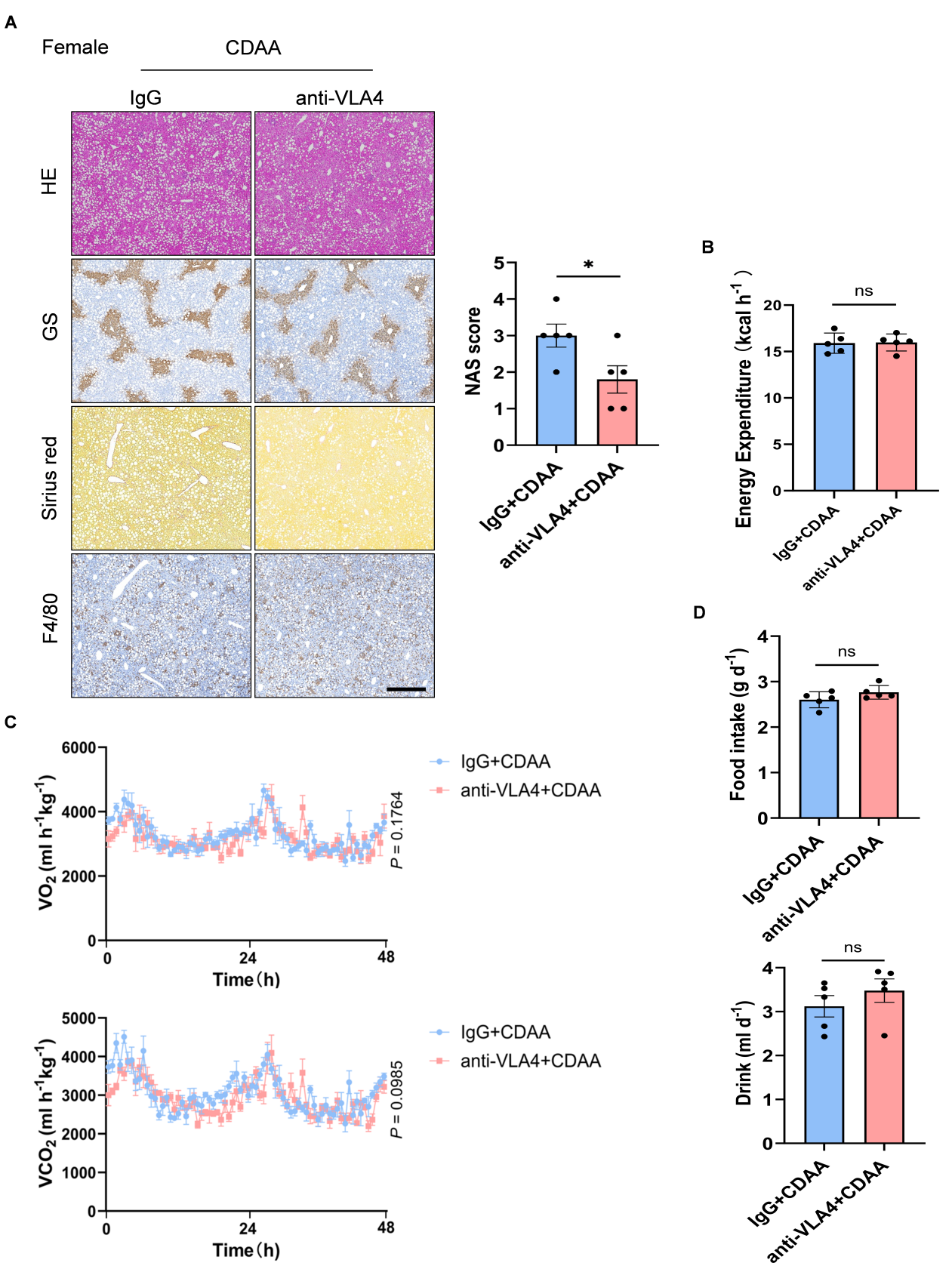


**Figure. S9. Anti-VLA4 treatment alleviates MASH phenotype in female mice and induces a therapeutic metabolic shift in male mice**

(A) Representative H&E, Sirius Red staining, and GS, F4/80 immunohistochemistry of livers from CDAA-fed mice and NAS of livers. Scale bars, 200 µm.

(B) EE in anti-VLA4-treated and control mice.

(C) Indirect calorimetry analysis in anti-VLA4-treated and control mice, monitored over a 48 h period.

(D) Average daily food and water intake in anti-VLA4-treated and control mice. Data are presented as mean ± SEM (n = 5). Statistical analysis was performed using two-tailed unpaired Student’s t-test (*p < 0.05, ns, not significant).


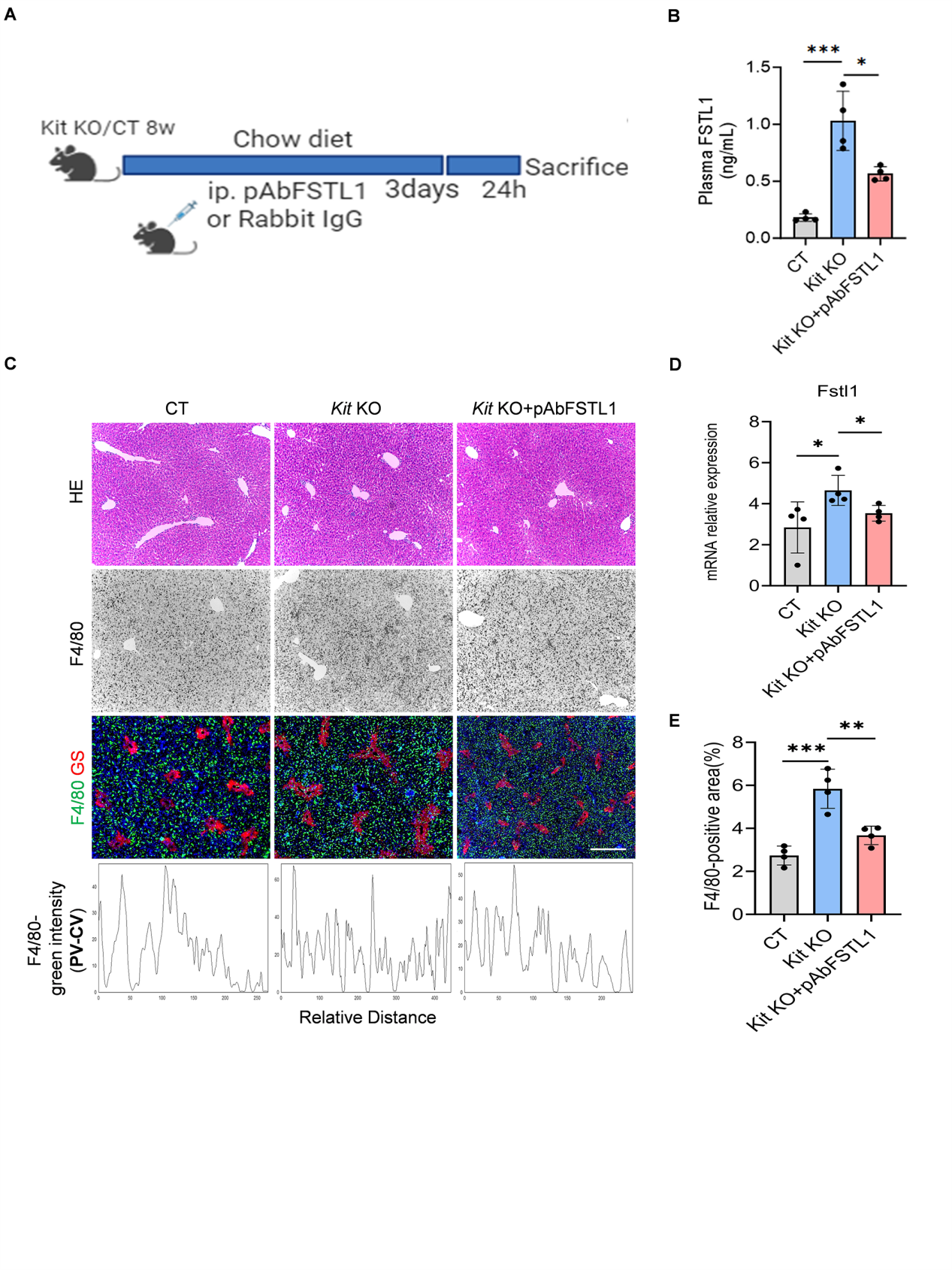


**Figure. S10. Antibody-mediated blockade of FSTL1 reduces macrophage infiltration in the liver of Kit KO mice**

(A) Schematic exerimental design.

(B) ELISA detection of serum FSTL1 levels in mice from each group.

(C) Representative H&E staining, F4/80 immunohistochemistry, and immunofluorescence of F4/80 (green) and GS (red) in liver sections from endothelial Kit KO and control mice. Scale bars; 200 µm.

(D) Detection of FSTL1 expression in the whole liver of mice from each group by quantitative PCR.

(E) Statistical analysis of F4/80^+^ area percentage in mouse liver. Data are presented as mean ± SEM (n = 4). Statistical significance was determined by Student’s t-test or multiple t-tests (*p < 0.05, **p < 0.01, ***p < 0.001).


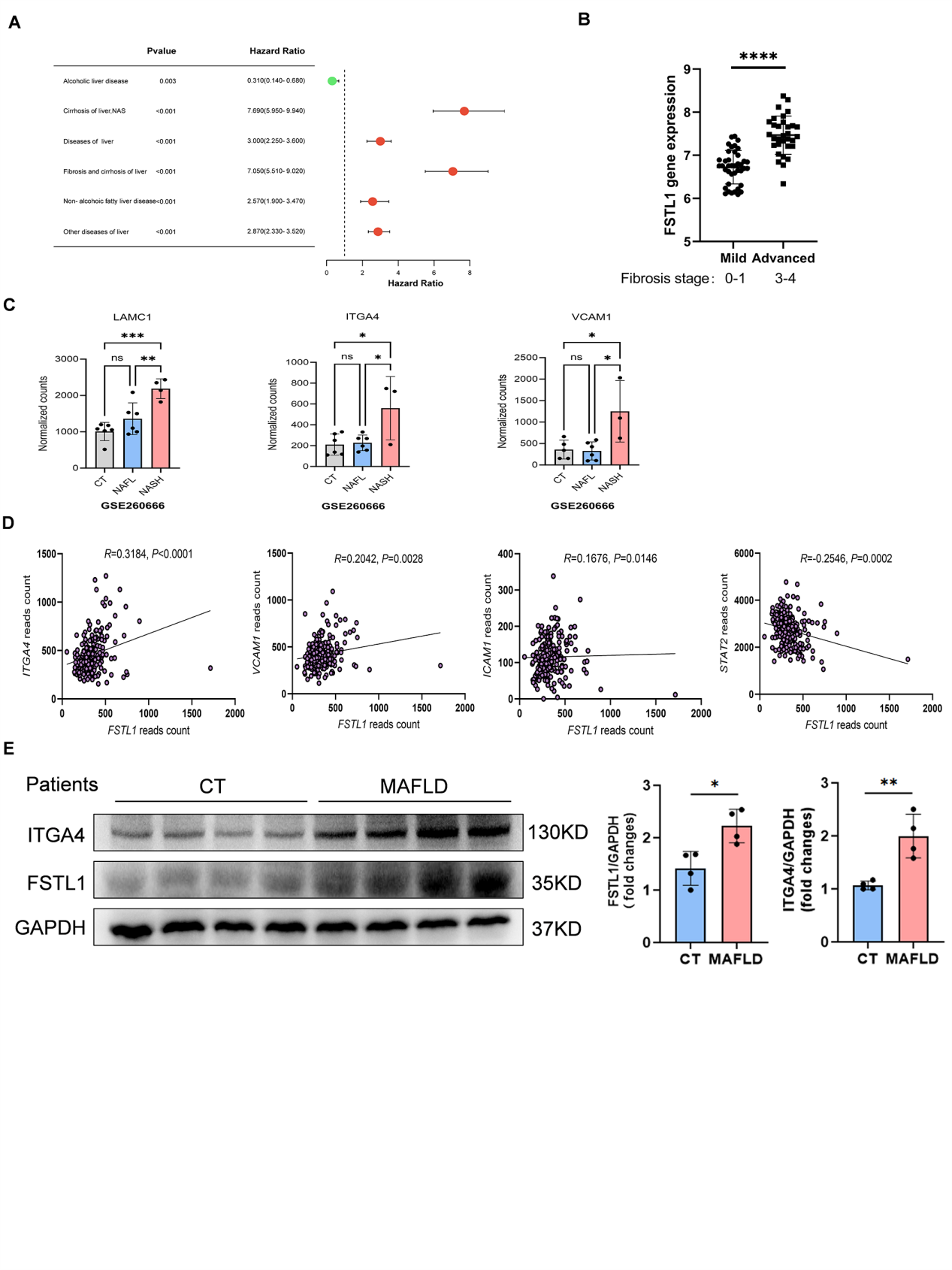


**Figure. S11. FSTL1 expression is elevated during human liver fibrosis and positively correlates with disease severity.**

(A) Forest plot of the association between FSTL1 and risk of various hepatic diseases.

(B) Quantitative analysis of FSTL1 expression across different fibrosis stages in patients from the Moylan et al. cohort.

(C) Analysis of a public dataset (GSE260666) showing expression levels of LAMC1, FSTL1, and VCAM1 in patients with MAFLD compared to controls.

(D) Spearman correlation analysis between the indicated molecules using data from GSE193084.

(E) Immunoblot analysis showing expression of liver-associated proteins in livers from MAFLD patients versus healthy controls(n = 4). Data are presented as mean ± SEM. Statistical analysis was performed using one-way ANOVA or pearson correlation analysis. (*p < 0.05, **p < 0.01, ***p < 0.001; ns, not significant).

**Supplementary tables**

**Table S1. Experimental models and genetically modified strains.**

| **Experimental models: Organisms/strains** | **Cat No.** | **Supplier** |
| --- | --- | --- |
| Cdh5CreERT | N/A | Generated in our laboratory |
| Kit-flox | N/A | Generated in our laboratory |
| Fstl1-flox | N/A | SHANGHAI MODEL ORGANISMS CENTER, INC. |

**Table S2. Diets and other materials.**

| **Other** | **Cat No.** | **Supplier** |
| --- | --- | --- |
| Chow diet | MD17121 | Medicience |
| Methionine and Choline Deficient L-Amino Acid Diet | A06071309 | Research Diets |

**Table S3. Reagents and materials used in this study.**

| **Chemicals, peptides, and recombinant proteins** | **Cat No.** | **Supplier** |
| --- | --- | --- |
| Recombinant human FSTL1 protein | HY-P70412 | MCE |
| Recombinant mouse FSTL1 protein | HY-P70859 | MCE |
| SCF Protein | RP01055 | Abclonal |
| BIO-1211 | HY-14126 | MCE |
| Phorbol 12-myristate 13-acetate | HY-18739 | MCE |
| Natalizumab | HY-108831 | MCE |
| Human IgG4 (S228P) kappa, Isotype Control | HY-P99003 | MCE |
| Anti-mouse/human VLA-4 (CD49d)-InVivo | A2162 | Selleck |
| Rat IgG2b isotype control-InVivo | A2116 | Selleck |
| Tamoxifen | T5648 | Millipore Sigma |
| Corn Oil | 8001-30-7 | Millipore Sigma |
| Collagenase IV | C5138 | Millipore Sigma |
| DNase I | 10104159001 | Roche |
| autoMACS® Running Buffer | 130-091-221 | Miltenyi Biotec |
| Optiprep | 1114542 | Axis-shield |
| CD146 (LSEC) Antibody | REA1064 | Miltenyi Biotec |
| CD117 Antibody | REA791 | Miltenyi Biotec |
| MS Columns | 130-042-201 | Miltenyi Biotec |
| C Tubes | 130-093-237 | Miltenyi Biotec |
| HBSS | BL560A | Biosharp |
| DMEM | C1885500BT | Gibco |
| ECM | 1001 | Sciencell |
| Trypsin-EDTA | 25300120 | Gibco |
| Lipofectamine2000 | 11668019 | Invitrogen |

**Table S4. Primer sequences for quantitative real-time PCR (qPCR).**

| **Gene** | **Forward (5’-3’)** | **Reverse (5’-3’)** |
| --- | --- | --- |
| IL-10(mus) | CCCATTCCTCGTCACGATCTC | TCAGACTGGTTTGGGATAGGTTT |
| IL-1β(mus) | GCAACTGTTCCTGAACTCAACT | ATCTTTTGGGGTCCGTCAACT |
| IL-6(mus) | TGGGGCTCTTCAAAAGCTCC | AGGAACTATCACCGGATCTTCAA |
| iNOS(mus) | CAGGGAGAACAGTACATGAACAC | TTGGATACACTGCTACAGGGA |
| Tnf-α(mus) | GGAACACGTCGTGGGATAATG | GGCAGACTTTGGATGCTTCTT |
| Arg1(mus) | CTCCAAGCCAAAGTCCTTAGAG | AGGAGCTGTCATTAGGGACATC |
| ARG1(hum) | TGGACAGACTAGGAATTGGCA | CCAGTCCGTCAACATCAAAACT |
| Cd80(mus) | ACCCCCAACATAACTGAGTCT | TTCCAACCAAGAGAAGCGAGG |
| Cd86(mus) | TGTTTCCGTGGAGACGCAAG | TTGAGCCTTTGTAAATGGGCA |
| Cd163(mus) | ATGGGTGGACACAGAATGGTT | CAGGAGCGTTAGTGACAGCAG |
| CD163(hum) | TTTGTCAACTTGAGTCCCTTCAC | TCCCGCTACACTTGTTTTCAC |
| Stat2(mus) | TCCGCTGTTCGCTATCTTGG | TGCGCCATTTGGACTCTTCT |
| STAT2(hum) | CCAGCTTTACTCGCACAGC | AGCCTTGGAATCATCACTCCC |
| Fstl1(mus) | TCTTGCCATTACTGCCACACA | CACGGCGAGGAGGAACCTA |
| FSTL1(hum) | GAGCAATGCAAACCTCACAAG | CAGTGTCCATCGTAATCAACCTG |
| Kit(mus) | GCCTGACGTGCATTGATCC | AGTGGCCTCGGCTTTTTCC |
| Itga4(mus) | GATGCTGTTGTTGTACTTCGGG | ACCACTGAGGCATTAGAGAGC |
| ITGA4(hum) | AGCCCTAATGGAGAACCTTGT | CCAGTGGGGAGCTTATTTTCAT |
| Acta2(mus) | GTCCCAGACATCAGGGAGTAA | TCGGATACTTCAGCGTCAGGA |
| Timp1(mus) | GCAACTCGGACCTGGTCATAA | CGGCCCGTGATGAGAAACT |
| Ccl2(mus) | TTAAAAACCTGGATCGGAACCAA | GCATTAGCTTCAGATTTACGGGT |
| Col1a1(mus) | GCTCCTCTTAGGGGCCACT | CCACGTCTCACCATTGGGG |
| Col3a1(mus) | CTGTAACATGGAAACTGGGGAAA | CCATAGCTGAACTGAAAACCACC |
| β-actin(mus) | GGCTGTATTCCCCTCCATCG | CCAGTTGGTAACAATGCCATG |

**Table S5. Antibodies used in this study.**

| **Antibody** | **Cat No.** | **Supplier** | **Purpose** |
| --- | --- | --- | --- |
| Kit | AF1356 | R&D | IF |
| F4/80 | 14-4801-82 | Invitrogen | IF |
| CLEC4F | AF2784 | R&D | IF |
| αSMA | ab124964 | Abcam | IF |
| FSTL1 | 20182-1-AP, ab223287  A15789 | Proteintech, Abcam  Abclonal | WB,IF |
| STAT2 | F0713 | Selleck | WB |
| pSTAT2 | AP0284 | Abclonal | WB |
| ITGA4 | 8440 | CST | WB, IF |
| GS | 11037-2-AP | Proteintech | IF, IHC |
| TUBLIN | GB15140 | Servicebio | WB |
| ACTIN | GB15001 | Servicebio | WB |
| GAPDH | GB15004 | Servicebio | WB |
| 7-AAD Viability Staining Solution | 420404 | Biolegend | FC |
| PerCP anti-mouse CD45 Antibody | 103129 | Biolegend | FC |
| PE anti-mouse F4/80 Antibody | 111603 | Biolegend | FC |
| Pacific Blue anti-mouse/human CD11b Antibody | 101223 | Biolegend | FC |
| FITC anti-mouse Ly-6C Antibody | 128005 | Biolegend | FC |
| PerCP anti-human TNF-α Antibody | 502923 | Biolegend | FC |
| Alexa Fluor 647 anti-human IL-1β Antibody | 511707 | Biolegend | FC |
| Donkey anti Rabbit Cy3 | GB21403 | Servicebio | IF |
| Goat Anti-Rabbit IgG H&L (AF594) | 550043 | Zenbio | IF |
| Donkey anti Rat Alexa fluo488 | A21208 | Invitrogen | IF |
| Donkey anti Goat Alexa fluo488 | A-11055 | Invitrogen | IF |
| HRP-Goat Anti-Rabbit Recombinant Secondary Antibody (H+L) | RGAR001 | Proteintech | WB |
| HRP-Goat Anti-Mouse Recombinant Secondary Antibody (H+L) | RGAM001 | Servicebio | WB |

**Table S6. Key commercial assay kits and reagents.**

| **Critical commercial assays** | **Cat No.** | **Supplier** |
| --- | --- | --- |
| Reverse transcription reagent | AG11706 | Accurate Biology |
| SYBR Green PCR Master Mix | AG11701 | Accurate Biology |
| Rabbit IHC kit | PV-9001 | ZSGB-BIO |
| ALT Assay Kit | C009-3-2 | Nanjing Jiancheng |
| AST Assay Kit | C010-3-2 | Nanjing Jiancheng |
| Mouse FSTL1 ELISA Kit | JL28982 | JONLNBIO |
| Human FSTL1 ELISA Kit | JL14965 | JONLNBIO |
| BCA Protein Assay Kit | 23227 | Thermofisher |
| Dual Luciferase Reporter Gene Assay Kit | RG027 | Beyotime |
